# Pre-Molecular Assessment of Self-Processes in Neurotypical Subjects Using a Single Cognitive Behavioral Intervention Evoking Autobiographical Memory

**DOI:** 10.3390/bs12100381

**Published:** 2022-10-05

**Authors:** Jorge Emanuel Martins, Joana Simões, Marlene Barros, Mário Simões

**Affiliations:** 1 Laboratory of Mind-Matter Interaction with Therapeutic Intention (LIMMIT), Faculty of Medicine, University of Lisbon, 1649-028 Lisbon, Portugal; 2 Center for Interdisciplinary Research in Health (CIIS), Faculty of Dental Medicine (FMD), Universidade Católica Portuguesa, 3504-505 Viseu, Portugal

**Keywords:** cognitive behavioral intervention, conscious experience, mental health profiling, neurophenomenology, neurotypical, self-concept, self-reflection, self-regulation

## Abstract

In the last 20 years, several contributions have been published on what concerns the conceptual and empirical connections between self-processes. However, only a limited number of publications addressed the viability of those processes to characterize mental health in neurotypical subjects with a normative pattern of neurodevelopment. Furthermore, even fewer experiments focused explicitly on the complexity of studying neurotypical phenomenal data. On the one hand, this normative pattern is commonly associated with mental health and a multifaceted self-concept and well-being. On the other hand, well-being is often related to a healthy cognitive life. However, how such intricate and complex relation between self-processes is established in neurotypical subjects requires further evidence. The novelty of this work is thus studying the first-person experience, which is correlated with the mental events aroused by a cognitive behavioral intervention. The prior methodology that led to the complete characterization of a neurotypical sample was already published by the authors, although the materials, the methods, the sample screening, and the sample size study required further explanation and exploration. This paper’s innovation is hence the phenomenological assessment of subjects’ self-regulation, which is used for mental health profiling, providing the basis for subsequent molecular typing. For that matter, a convenience sample of 128 (19–25-year-old) neurotypical young adults, healthy university students at the University of Lisbon, non-medicated and with no serious, uncontrolled, or chronic diseases, are characterized according to their cognitive functioning and self-concept. The procedure comprised (i) a mental status examination (psychological assessment) and (ii) a psychological intervention, i.e., a single cognitive behavioral intervention (intervention protocol). The psychological assessment was a standardized and structured clinical interview, which comprised the use of 4 psychological scales complementary to the classical Mental Status Examination (MSE). The intervention protocol applied a combined exercise of psychophysical training and autobiographical-self memory-recalling. The results permitted identifying and isolating four different subgroups (self awareness, self consciousness, reflective self, and pre-reflective self) in neurotypical subjects with discrete self-processes. The outcome of this study is screening four different aspects of self-reflection and the isolation between various forms of self-directed attention and their interconnections in these four mental health strata. The practical implication of this study is to fulfill an a priori pre-molecular assessment of self-regulation with separate cognitive characteristics. The reliability of these mental strata, their distinct neurophysiology, and discrete molecular fingerprint will be tested in a future publication by in silico characterization, total protein profiling, and simultaneous immunodetection of the neuropeptide and neuroimmune response of the same participants.

## 1. Introduction

A phenome is the sum of phenotypic characteristics of an organism and reflects the interaction of the complete genome with the environment [1]. The phenome is usually composed of functional states and traits [2] and can typically be studied by its subphenomes. Sexually dimorphic species, like humans, have a phenome comprised of subphenomes [3] or functional subgroups. These functional subgroups are commonly associated with specific molecular pathways and the product of clustered phenotypes sharing common characteristics [4]. By definition, a phenotype is a state of an organism resulting from interactions between genes, environment, and molecular mechanisms [5], expressing physical, behavioral, and psychological traits or functioning [6]. A healthy neuropsychophysiological functioning [7] may be described, by molecular biology, as a healthy phenome, i.e., the healthy set of all phenotypes expressed by an organism, representing the total sum of its phenotypic traits [8]. A phenotypic trait is a distinct variant of a phenotypic characteristic of an organism that may be inherited and determined environmentally [9]. 

Measurements of brain response are based on the premise that different neurophysiological states accompany different cognitive states. A state alludes to altered sensory, perceptive, and self-referential awareness, whereas a trait refers to lasting neurological and mental changes [10,11,12]. The cognition of the healthy individual may reflect a more focused and aware mental state and trait. These states and traits are characterized by good physiological functioning and neuroplasticity, possibly leading to new brain correlates [13,14]. This research’s main aim is to study the first-person experience of neurotypical subjects, defined as healthy individuals demonstrating a normative pattern of neurodevelopment [15]. The secondary objective is to isolate distinct mental events aroused by a cognitive behavioral intervention, increasing awareness and wakefulness, by a cognitive exercise of psychophysical training and autobiographical-self memory-recalling [16]. Psychophysical training comprises exercises concentrating attention on body parts, mental images, or breathing to regulate emotions and behavior [17,18]. As an expected result, this cognitive exercise created separate cognitive states, from less focused on self-concepts to more aware of self-regulation [17], thus, permitting the mental profiling of individuals without a neuropsychiatric condition by self-report measures.

Those states and traits induce long-term changes in self-consciousness, observed both in high- and low-level cortical representations [19,20]. Moreover, it has been shown that those cognitive states have an essential role in self-knowledge and self-concept [21], which is defined as “one’s self-identity, a schema consisting of an organized collection of beliefs and feelings about oneself” [22]. Psychological interventions can induce these functional changes, which modulate conscious experience and self-referential awareness. Self-referential awareness refers to the capacity of a human being to interpret incoming information concerning himself, using his self-concept as a background for new information. Both natural and induced changes have been described in the literature [20,23]. The natural ones correspond to trait mindfulness, whereas the induction ones involve a second individual that leads the induction and facilitates the achievement of a desired mental state or trait [19,20].

These are well-studied and have been recognized as effective clinical tools [24]. These referred techniques are also vehicles for inducting subjective states of happiness and well-being [25,26]. These have been described as constructs associated with health in neurotypical subjects. One way to attain this cognitive state and trait is through a cognitive behavioral intervention [27]. Trait mindfulness is described as sustainable awareness aiming at a non-reactive consciousness [28,29,30,31]. Trait mindfulness is a physiological state and trait distinct from exclusive pre-reflective or reflective self-experience, or self-reflection [31,32,33,34,35,36]. Trait mindfulness is known to promote physical and mental well-being with positive emotional traits [37,38] and modulates the limbic-neocortical emotional systems connectivity [31,32,39]. This phenome state might be induced with a cognitive behavioral intervention [27], which has two interlinked components:

(1) a sustained reflective self, to the present moment (awareness), related to the quality of the conscious experience in the very moment of its occurrence [40,41].
(2) an open and acceptant attitude towards the experience (arousal), the pre-reflective self-experience.

This specific mental state [42,43] concedes a continuous process of internal and external self-monitoring (monitor self-presentations, expressive behavior, and nonverbal affective displays. It is defined as a personality trait that refers to an ability to regulate behavior to accommodate social situations) and self-regulation [17], which has been shown to lead to neuropsychophysiological changes [25,26,44,45]. Until now, no valid and reliable instrument probes into healthy individual cognition, but only into singular functional subconstructs (e.g., well-being or *eudaemonia* [46,47]. Nevertheless, different publications have altogether probed into subconstructs exclusive to mental health functioning in neurotypical subjects [48]. For a clinical evaluation of mental health and experiential variables, self-report measures are usually used, assessing those state and trait variables. This paper proposes a method used as a clinical strategy for profiling those neurotypical subjects. 

“A plurally constituted approach can be rigorously formulated to accommodate qualitative analysis and quantitative measurements” [49]. Physiological mechanisms have described that phenomes composed of strong emotional states also modify the protein biomarker profile [50]. These are examples of quantitative measurements of potential proteins obtained by capillary electrophoresis, an analytical technique that separates ions based on their electrophoretic mobility with an applied voltage. The electrophoretic mobility is dependent upon the charge of the molecule, the viscosity, and the atom’s radius [51]. In addition, peptides such as encephalins might also serve as additional parameters for influencing and reflecting baseline states and traits of cognition, as measured by a phenomics approach [52]. Phenomics is thus the study of sets of traits belonging to an organism [53] by systematic measurement and analysis of qualitative and quantitative traits, including clinical, biochemical, and imaging methodologies, for the refinement and characterization of a given phenotype [54].

To our knowledge, no research seems to have combined analytical techniques with changes in physiological states resulting from the self-conscious experience [55]. Self-conscious experience concerns, in our experiment, first-personal thinking and its representational content, but also the various forms of sensory experience. This paper aims to provide the psychological analysis or basis for a subsequent physiological approach through a molecular biomarker analysis. Our research hypothesis is that the pre-molecular assessment of the self-processes of the 128 neurotypical subjects conducted in this experiment might be a genuine and interesting framework for the objective research of self-consciousness. 

As the sensitivity of assessing central nervous system conditions is becoming increasingly possible [56] by phenomics techniques, our research question remains around their sensitivity to detect functional protein networks directly correlated to specific phenotypic states and traits. The problem resides if these different functional mental health states might be objectively assessed through a total protein profile analysis in a phenomics study design. 

To advance with our already published results, combining a psychological assessment with a molecular measurement of protein profiles, in this experiment, we fully describe the procedure adopted, viz., a cognitive-behavioral intervention [57,58]. 

### Self and Cognitive Behavioral Intervention

The idea of self [59] is understood as the capacity “to order my own thoughts and my own life, to use reason as an instrument to control and order my own life”. Thus, the self is the internalization of our experience [60]. Studies conducted on university students demonstrated that self-training derives from cognitive modeling and imagery rehearsal as the paradigm employed in cognitive behavioral interventions. As behavior therapy techniques were altered to incorporate cognitive factors, the likelihood of achieving metacognitive access increased [57,58], i.e., the awareness or analysis of one’s learning or thinking processes [61].

According to Thompson’s study [62], one sort of cognitive incorporation was possible by focused attention interventions, such as the one used in this study. These interventions are well developed and achieved in cognitive behavioral intervention, where the subject attends to characteristics of experience that are typically ignored and/or neglected [63]. Likewise, the participant may observe features of himself that he infrequently does [14,62,64]. Thus, focused attention interventions can be comprehended as some phenomenological training, which permits the scientific analysis of the mind [65]. This intervention aims to enable participants to relate to their thoughts as objects of awareness, i.e., as “mental events” [27]. Henceforward, the psychological intervention, i.e., a cognitive behavioral intervention, used in this study is described as a methodical process of familiarizing the subject with a present character of a mental event, cultivating his capacity for sustained and attentive awareness [14,66]. 

## 2. Materials and Methods

A qualitative study is performed, in which a cognitive behavioral intervention is used as the experimental procedure. 

### 2.1. Study Design

The study is a non-representative, descriptive study with a two-grouped, four-setting, single-blinded and post-test design. Please refer to Hipólito and Martins’s study [49] for a complete description of the study design. The experiment is controlled, multicentered, and approved by the Ethics Committee of the Faculty of Medicine, University of Lisbon. 

The method comprehends a descriptive research strategy [67] combining:

(i) psychological intervention, i.e., the Intervention Protocol;
(ii) clinical assessment, i.e., the Assessment Protocol.

Thus, the investigation was performed in two groups: the intervention group exposed to a cognitive behavioral intervention evoking a more conscious experience, and a control group attending a routine university class. After the Intervention Protocol, all the subjects (experimental procedure and sham intervention) were clinically examined concerning the: (1) process and content of thought and perception; (2) mood and affects; (3) cognitive awareness and attention; (4) insight and judgment. The subjects were asked to conduct this qualitative evaluation using the four psychological scales summarized in 2.4.

In this paper, we advanced with the previously published methodology [49], using the same participants, basing our methods on the stratification of neurotypical subjects into subclinical groups [7,14,20,33,34,36,68,69,70,71,72,73,74,75]. 

### 2.2. Statistical Population and Sample of the Study

To conduct the experiment, a convenience sample of 128 healthy volunteers (19 to 25 years old), neurotypical young adults (attaining a proportion of 50% male), was recruited from the University of Lisbon (Faculty of Medicine, Faculty of Economics, Faculty of Sciences, and The School of Social and Political Sciences).

The statistical population studied is non-medicated healthy university students from the University of Lisbon without serious, uncontrolled, or chronic diseases. We sampled our subjects from a convenient and readily available population from the University of Lisbon. We used this method, i.e., grab sampling, because we could not obtain a list of all the students at the University of Lisbon and include them in our descriptive study type. One of the main limitations is that the method cuts out a large part of our population.

#### Power Analysis and Sample Size Study 

Please refer to Hipólito and Martins [49] for a complete statistical strategy and sample size estimation. The primary outcome of this work is to observe if there is a molecular correlation to mental health stratification. Hence, power analysis [76] was used to “calculate the minimum sample size required so that one can be reasonably likely to detect an effect of a given size”. 

The sample size study is thus supported by the previous preliminary results of the experimental group [55]. Sample size determination [77] was done to choose the “number of observations to include in our statistical sample”.

These results were obtained before advancing with the mental health stratification. Consequently, it was aimed to test if there was a significant change in the protein profile in a neurotypical sample. The sample size was an essential feature of this empirical study in which the goal was to make limited inferences about a population from a neurotypical sample. In practice, the sample size used in this exploratory study was ultimately determined by the high cost of the molecular analysis and the need to offer sufficient statistical power still. 

**Figure 1:**
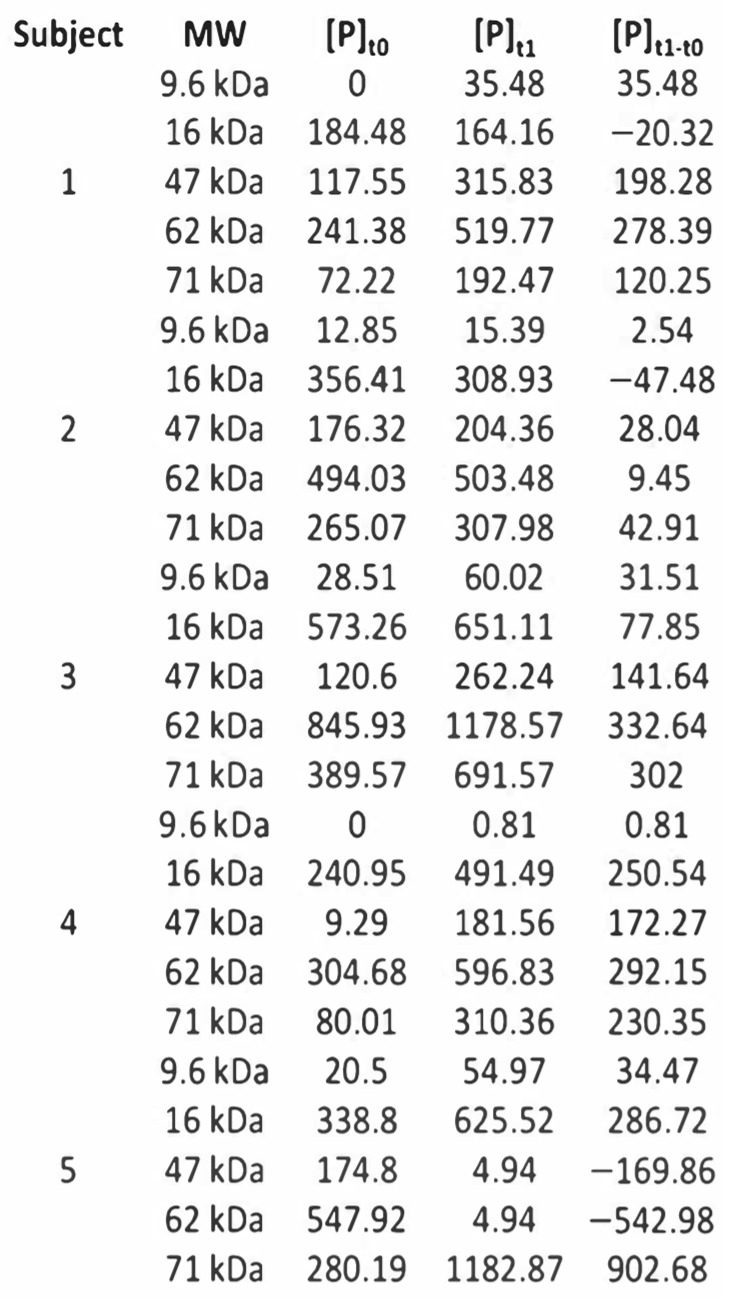
Power analysis and sample size study, using the Δ[P] after-before (experimental procedure). The five preliminary subjects’ protein concentrations and change/variability (ng/µL) are presented. Five MWs of interest were used to estimate a potency (T-Student) of 80%, a significance level of 5%, and an effect size of 40%.

Figure 1 shows the quantitative data of the tested Molecular Weights (mass of a given molecule, measured in Daltons (Da) [78] (MWs) used to evaluate significant changes in the protein profile by capillary electrophoresis. Capillary electrophoresis method allowed to measure the concentration (ng/µL) of the five most significant ranges of MWs in the preliminary study: 9.6 kDa, 16 kDa, 47 kDa, 62 kDa, and 71 kDa, before and after the experimental procedure. 

The following approach was designed and used to calculate the adequate sample size for this study: 

First, the difference Δ[P] after-before (experimental procedure) of the protein concentration (ng/µL) was calculated, i.e., the protein change/variability (ng/µL). Secondly, based on the change/variability (ng/µL), it was then considered a potency (T-Student) of 80% and a significance level of 5%. Thirdly, an effect size was chosen (in research, the effect size is the magnitude of the difference between groups [79]), corresponding to 40% of the estimated value for the standard deviation of the variation of the MWs. 

Considering this statistical option, which corresponds to a medium Cohen’s effect size, an appropriate sample size for each group (experimental and control) of 64 elements were found. Consequently, the sample was initially separated into two independent groups: (a) Experimental group (experimental procedure) (64 subjects); and (b) Control group (sham intervention) (64 subjects). 

### 2.3. Screening

#### 2.3.1. Inclusion and Exclusion Criteria

The following criteria screened the neurotypical students:

(a) Inclusion criteria: (1) 19–25-year-old young adults; Male/Female; (2) Cultural background: university students; (3) Normal body mass index (BMI) and (4) Non-medicated except for birth control pills;
(b) Exclusion criteria: (1) Serious physical illness or uncontrolled kidney, liver, lung, heart, musculoskeletal, rheumatologic, metabolic, neurological, or psychiatric disorders ; (2) Severe chronic or terminal disease, which might affect the Central Nervous System (CNS) or the Peripheral Nervous System (PNS); (3) Pregnant or breast-feeding women; (4) Abuse of alcohol or addictive substances, prior to the experience.

#### 2.3.2. Sample Screening and Group Characterization 

For preliminary sample screening and group characterization of this experiment, please refer to Hipólito and Martins [49]. As already published, two different groups were created: a) the experimental group, practicing a demanding attentional and emotional task with autobiographical memory recalling; and b) the control group, attending a regular class. Afterward, those two groups were requested to qualitatively assess their personal experience using a Psychological Assessment (Section 2.4.1). 

In this experiment, those two groups were thus stratified into the top and bottom phenomes, depending on their total scores on the Psychological Assessment, resulting in 4 subgroups:

(i) The top phenome of the Experimental group;
(ii) The top phenome of the Control group;
(iii) The bottom phenome of the Experimental group;
(iv) The bottom phenome of the Control group.

**Figure 2:**
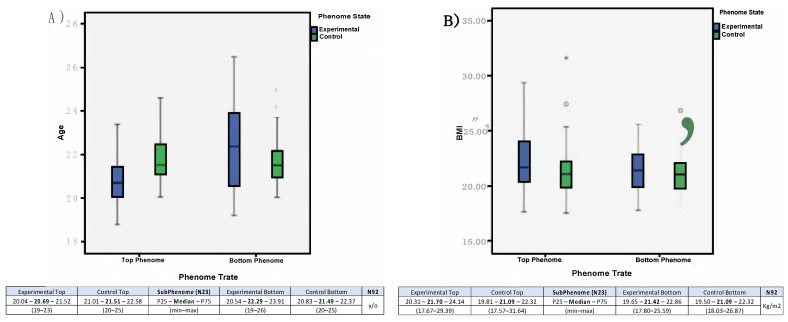
Biometric data of the neurotypical sample. In (**A**), a boxplot (P25, median, P75, minimum and maximum) of the sample’s age in the four subphenomes (Experimental Top subgroup, Control Top subgroup, Experimental Bottom subgroup, and Control Bottom subgroup) is presented. In (**B**), a boxplot (P25, median, P75, minimum and maximum) of the sample’s BMI (Body Mass Index), in kg/m^2^, for the same four subphenomes is presented.

This stratification assigned 92 subjects, as it comprehended the use of the higher (Top) and lower (Bottom) tercile of the sample (further explained in Results and Discussion), and an additional 10% of subjects. These final four subgroups (profiles), with an *n* = 92, were tested for comparability statistics and biometric data, possibly confounding variables, and were considered comparable for:

(a) Age (Figure 2A) (P25; median; P75) and overall (*n* = 92) mean = 21,495;
(b) Gender mean = 50% males and SD = 0;
(c) BMI (kg/m^2^) (Figure 2B) (P25; median; P75).

#### 2.3.3. Sampling, Allocation, Randomization, and Blinding

As we used a convenience sample, we applied a non-probabilistic sampling, which did not include a random selection from the whole list of university students at the University of Lisbon. Our sample of 128 neurotypical subjects is therefore not representative of the entire statistical population. 

The allocation of the neurotypical young adults was based on the students attending the different lectures. Thus, the subjects were arbitrarily assigned to an experimental group or a control group, (i) according to the randomization of the weekly theoretical-practical classes for each course of each faculty, and (ii) depending on the students who were enrolled in one or another class. At any educational institution and by parallel assignment (1:1), students in Tuesday’s class were allocated to the experimental group (experimental procedure), and students in Thursday’s class were allocated to the control group (sham intervention). 

Randomization was done mainly: (i) to balance the effect of internal conditions associated with the students attending courses at the various colleges of the University of Lisbon, and (ii) to program the logistics of collecting data. Thus, the assignment was parallel, the allocation randomized, and the Experimental and Control groups, with a 1:1 allocation, were strictly conserved. 

The blinding was maintained in the Intervention and Assessment protocols. As enrolled in the study, subjects received information and filled out the informed consent. The confidentiality was maintained following the Biobanco-IMM and SalivaTec-CIIS defined confidentiality protocols. 

Biobanco-iMM CAML is integrated into the Academic Medical Center of Lisbon (CAML) and includes biological samples (from surgery, biopsies, blood samples, etc.) voluntarily donated with permission for preservation and future use in biomedical research. SalivaTec-CIIS focuses his research on the molecular analysis of biomarkers, curating and integrating molecular data focused on salivary diagnostics.

Single blinding was maintained on the overall mental health and molecular stratification. However, in the execution of the molecular assessments, a triple blind was guaranteed, as neither the researcher, the analyst, nor the statistician knew the sample screening.

#### 2.3.4. Limitations of Sampling and Bias

We were careful not to generalize the experiment’s results to the statistical population, avoiding the possibility of under- or over-representation of the population. Our results thus have a selection bias, which may be reanalyzed and reproducible but not replicable or generalizable to the larger population of healthy university students aged from 19 to 25 years old. Our biggest concern with our convenience sampling is dependency, as our sample’s dependent variables may be connected, and this dependency interferes with some of our future statistical analyses. Although our sample does not represent the population of university students at the University of Lisbon, it enables a descriptive type of study. We avoided having: (i) volunteer bias, since the subjects who volunteered for the experiment all belonged to the University of Lisbon; (ii) allocation bias, since we used an appropriate randomization strategy in our convenience sample, avoiding marked and systematic differences between experimental and control groups; (iii) assignment bias, as we maintained a rigorous assignment process. However, due to the nature of the intervention protocol, participants knew in advance which group they were being allocated to, which created an inevitable bias.

### 2.4. Procedure

The authors already published the procedure that led to the characterization of the neurotypical sample [49]. However, this paper comprises the stratification strategy and details the materials (Psychological Assessment) and the methods (Intervention Protocol) used for mental health stratification. The procedure included a mental status examination and a cognitive behavioral intervention in the 128 neurotypical young adults sample. More specifically, this paper presents the clinical strategy for the adequate profiling of mental status. This strategy describes (i) the psychological intervention, extending the use of the cognitive behavioral intervention, but also (ii) the mental health profiling of the 128 subjects.

#### 2.4.1. Materials Used in the Psychological Assessment

The materials that were used in the Psychological Assessment are described below. These materials were applied in a standardized and structured clinical interview using the following four psychological scales. Those materials were used for the classical Mental Status Examination (MSE) [80] but in a sample of neurotypical subjects. This allowed the primary to compare (a) the experimental group and (b) the control group and, additionally, to investigate their personal experience concerning its psychological features [49]. 

The qualitative and quantitative psychological evaluation included subjective descriptions of the subjects and described their mental state. This standardized and structured clinical interview is inspired by the MSE, which “derives from an approach known as descriptive phenomenology” [81,82] developed by Karl Jaspers. As theorized by Jaspers, but here applied to a neurotypical sample of young adults, this standardized and structured clinical interview assumes “that the only way to comprehend a subject’s experience is through his description” [83].

As published in Hipólito and Martins [49], self-report measures were applied to a sample of 128 neurotypical subjects. The following psychological validated scales were used: (i) *Abnorme Psychischer Zustände* States of Consciousness (APZ) [84]; (ii) Subjective Happiness Scale (SHS) [85]; (iii) Mindful Attention Awareness Scale (MAAS) [86]; (iv) Self-consciousness Scale—Revised version (SCS-R) [87].

(i) *Abnorme Psychischer Zustaende* States of Consciousness (APZ) [88], termed in English the Altered States of Consciousness Rating Scale, was used to evaluate both the process and content of thought, as well as perception. The thinking process describes the stream and rate of ideas and how they flow and are connected. Hence, “thought process refers to the quantity, tempo (rate of flow) and form (or logical coherence) of thought” [89]. The content of thought refers to the themes that occupy the subject’s thoughts and perceptions, i.e., the qualitative properties of thinking and its intensity, salience, and associated emotions [90]. A perception in the context of this evaluation “is the organization, identification, and interpretation of sensory information to represent and understand the presented information or environment” [91]. The items in the scale fundamentally estimate the alterations of thought, possible intense emotional responses, bodily schema changes, perceptual changes, and meaning alterations, which are characteristically aroused during cognitive behavioral interventions.
(ii) Subjective Happiness Scale (SHS) [92] was used to assess mood and affect. Trzepacz and Baker [80] described the mood as “a person’s predominant internal state at any one time” and the affect as “the external and dynamic manifestations of a person’s internal emotional state”. The mood is thus evaluated as the prevalent subjective state, i.e., the emotional type (euthymic, dysphoric, euphoric), and affect as the congruency, intensity, and mobility with the global subjective experience and thought content. As fully described in Hipólito and Martins [49], “two items evaluated absolute and relative personal experience; two other items evaluated absolute and relative external experience”. Thus, SHS evaluates the “constructs of subjective happiness and well-being, but also its stability [47]. In the context of this experiment, SHS intends to measure the self- and non-self perception of happiness, which can be stable or labile [93].
(iii) Mindful Attention Awareness Scale (MAAS) [37] was applied to evaluate cognition, and more specifically, the level of consciousness, alertness, and attention, i.e., “the awareness of, and responsiveness to the environment” [94]. MAAS evaluates the ability to focus, sustain and appropriately shift mental attention [38,49]. It explains that MAAS probes into a “unique quality of consciousness related to, and predictive of, pre-reflective self-experience”. Thus, it evaluates the subjects’ open awareness of the present, i.e., the exercise of immediate and short-term memory.
(iv) Self-consciousness Scale—Revised version (SCS-R) [95] was applied to measure insight and judgment. Insight refers to the participant’s understanding of his mental state and awareness of his situation [96]. For Martin and Hickerson [97], the judgment indicates “the ability to make reasoned and responsible decisions to preserve one’s and others’ wellbeing” [47]. Hence, this assessment considers the participant’s impulsiveness, social cognition, and self-awareness [98]. SCS-R evaluates thus private and public self-consciousness. This component or domain evaluated in a classical MSE is considered relatively stable over time and arises from sustained consciousness, long-term memory, and the capacity for abstraction and interpretation [17].

The contents and constructs evaluated by each scale are summarized in Hipólito and Martins [49]. An illustration of the variables and constructs is subsequently shown for each self-report measure to better understand the items evaluated on each psychological scale. Moreover, the complete materials are fully presented in Appendix A**.** Case Report Form.

For example, in the APZ scale, the central four constructs assessed were (i) the content of thought and associated mood; (ii) cognitive processes; (iii) auditory-visual reformulations or changes in the meaning of percepts; and (iv) general consciousness alteration [99]. Dittrich [100] defined “three primary and one secondary etiology-independent dimensions of the APZ”. For Dittrich [100], the three primary dimensions were designated as “oceanic boundlessness, dread of ego dissolution, and visionary restructuralization”.

The Psychological Assessment for the mental status examination considers these three primary dimensions. The secondary scale, i.e., the altered global states of consciousness scale, was used not only in the Psychological Assessment but also for a sequential publication of this work for mental health profiling. Twenty-three binary items constitute APZ’s secondary scale. In our neurotypical sample, APZ was used to evaluate “sensory gating and its relation to cognitive alterations” [101,102], e.g., concentration; but also the “individual’s openness to a variety of cognitive, perceptual, and imagistic experiences by vivid imagery” [103,104].

Six items constitute the SHS scale on a [0; 7] scaling, and the central construct assessed was self and non-self experiences of happiness. SHS assesses the balance of positive and negative emotions experienced over a particular period and the capacity to judge the overall quality of life. This judgment is “likely not equivalent to a simple sum of the subject’s recent levels of affect and satisfaction with life, but indicative of an overall affective and emotional perception” [92,93]. 

The MAAS scale comprises 15 items on a [1; 6] scaling, and the primary construct evaluated was open-awareness experiences. MAAS measures private self-monitoring and cognition constructs and is targeted for cognitive behavioral interventions and designed for nonclinical samples, the case (intervention and sample) of this study. This assessment probes the effective regulation of emotionality and attention capacity to present internal state experiences [105].

The SCS-R scale is composed of 23 items on the five-point Likert scale, and the central construct evaluated was private and public self-consciousness. SCS-R scale assesses the constructs of self-reflection and self-concept [106,107]. This assessment probes into the effective regulation of internal state awareness and the capacity for self-presentation and introspection, i.e., insight and judgment.

#### 2.4.2. Methods Used in the Intervention Protocol

The method, or Intervention Protocol, comprised an experimental procedure with a cognitive behavioral intervention. Following the paradigm of Kavanagh and colleagues [108], the objective was to “explore the impact of preventive interventions on inequalities in young people’s mental health” [48]. This study used this method to study neurotypical young adults by applying a combined relaxation and suggestion intervention to evoke positive past memories. It is well established that a variety of cognitive-behavior interventions, as shown by Dunning and colleagues [109] meta-analysis of randomized controlled trials, ultimately improves the reduction of neuropsychiatric symptomatology in subjects with clinical risk factors or existing symptoms. These interventions include mental imagery and are not only effective at addressing “symptoms such as pain, anxiety, nausea, and insomnia but also known to improve mental health and well-being” [47,110].

##### Cognitive Behavioral Intervention

For Khema and colleagues [111], trait mindfulness is “a focused and stable state of concentration”, which is extensively investigated by neuroscience and used in cognitive behavioral interventions. As stated by Leung and colleagues [112], such cognitive behavioral interventions have been shown to “play a physiological role in inducing neuroplastic changes in amygdala activity”, as well as preventing age-related changes [113] in cognitive functioning. In addition, for Xu and colleagues [114] and Hoge and colleagues [115], cognitive behavioral interventions appear to stimulate changes in attentional focus. Physiologically, these cognitive-behavioral interventions may be directed toward the short-term goal of stimulating the dopaminergic reward system [25,26]. Henceforth, in this study, a cognitive behavioral intervention was implemented in the experimental group to stimulate this possible feedback loop by switching attentional focus.

##### Description of the Intervention Protocol

**Figure 3:**
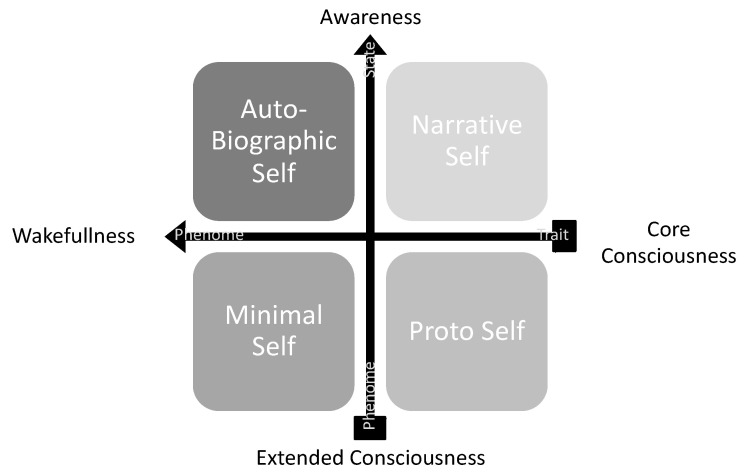
Neurophenomenological sampling and subgroup identification: constructs of self. A two-axis plot represents the Intervention Protocol, and the types of self [117]: Proto Self, Minimal Self, Narrative Self, and Autobiographic Self, stimulated in the two phases of the experimental procedure. On the horizontal axis, the progression of the mental trait is plotted, stimulating core consciousness to a more wakefulness trait. On the vertical axis, the progression of the mental state is plotted, enabling extended consciousness to a more awareness state. This figure was created following the work of the authors Hipólito and Martins [49].

The Intervention Protocol (experimental procedure and sham intervention) was carried out in one single session, on Tuesdays or Thursdays, performed between 09:45 (a.m.) and 10:30 (a.m.). The experimental procedure is a structured psychological intervention, i.e., a single cognitive behavioral intervention [27]. It was applied in a psychotherapeutic setting [24,44,55,116] with a total duration of 45 min. The research aim was to increase awareness and wakefulness (Figure 3) through a cognitive exercise of psychophysical training and autobiographical-self memory-recalling [16]. The intervention script is available in Appendix B. Verbal protocol of the experimental procedure, and is based on the publications of Simões and colleagues [21,44,116]. It was initially tested in the pilot study of this experiment [55] and as the experimental procedure of this work. 

**Figure 4:**
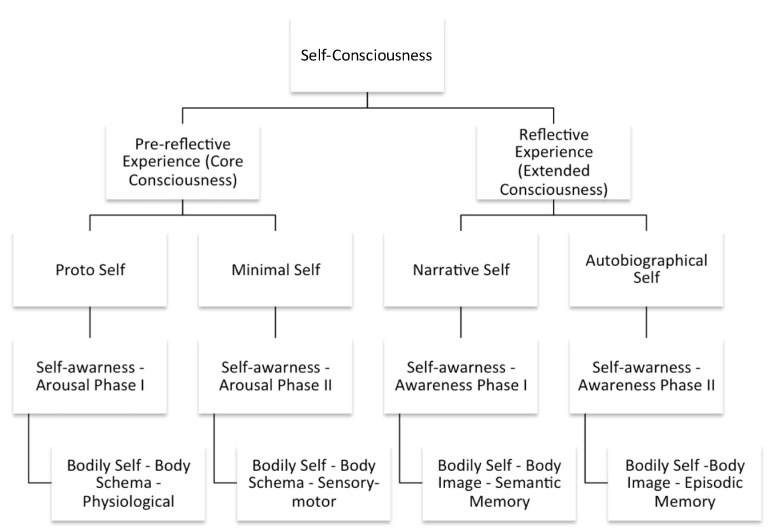
Fluxogram of the neurophenomenological method. A fluxogram is presented to systemize the intervention Protocol and the two phases of the experimental procedure: the Arousal Phase and the Awareness Phase. The correspondence to the bodily self and body schema [118] induced in each phase is represented. This figure was created following the work of the authors Hipólito and Martins [49].

This exercise helped stratify the mental health state and traits of the neurotypical subjects in the study by somatic, emotional, and cognitive regulation. The procedure was divided into two main phases, and the outcomes are further described in the flow chart in Figure 4.

The 1st phase, the *Arousal Phase* [69,74], is a conditioning procedure, associating neutral stimuli with biologically meaningful outcomes [119]. With a duration of 15 min, it aims to modulate arousal or the level of consciousness. The exercise consisted of a series of directed verbal cues to enhance somatic focus, i.e., on breathing patterns (e.g., “Concentrate on your inhalation.”) and postural positioning (e.g., “Feel your right forearm.”). According to Gallagher [118], this phase may help the subject be aware of his bodily self and body schema. The somatic focus used in this Intervention Protocol is defined, by Gallagher [118], as (i) physiological self-awareness and (ii) sensory-motor self-awareness, as illustrated in Figure 4. 

**Figure 5:**
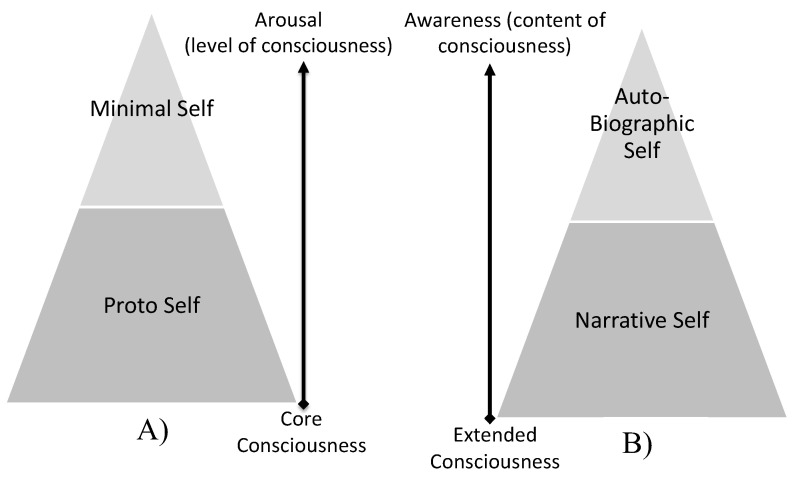
Conceptual analysis of the intervention Protocol: (**A**) Arousal phase and (**B**) Awareness phase. A pyramidal hierarchization is represented for each phase. In (**A**) the Arousal phase, the level of consciousness is plotted. In (**B**) the Awareness phase, the content of consciousness is plotted.

The outcome of this preliminary 15 min procedure is stimulating a pre-reflective self-experience [16], modulating what Damasio and Meyer [117] described as core consciousness and/or the proto and minimal self [120], as illustrated in Figure 5A. 

The 2nd phase, the *Awareness Phase* [121,122], is an expectancy procedure [123,124] which is the process of conscious and conceptual identification of the future occurrence of an event [119]. It is thus a subclass of predictive processes, which may be conscious or unconscious [119]. With a duration of 30 min, it aims to modulate awareness or the content of consciousness. The exercise consisted of a series of directed verbal cues to enhance a memory focus, i.e., sequential memory recalling (e.g., “Remember your first day of school”; “Remember your favorite holiday”, “Remember your last birthday”). These were linked to meaning-making and reflexive emotional verbal prompts (e.g., “How good was it?”). 

According to Gallagher [118], this phase may help the subject become aware of his self-body image. The memory focus used in this Intervention Protocol is defined, by Gallagher [118], as (i) semantic memory self-awareness and (ii) episodic memory self-awareness, as illustrated in Figure 4. The outcome of this 30 min exercise is stimulating a reflective self-experience [16], modulating what Damasio and Meyer [117] described as extended consciousness and/or the narrative and autobiographical self [120], as illustrated in Figure 5B. Therefore, based on this Intervention Protocol, two separate cognitive tasks, with a duration of 45 min, were established: (a) the experimental procedure and (b) the sham intervention. 

On the one hand, in the experimental procedure (a), the Intervention Protocol was used in the Experimental group and comprised both arousal and awareness phases. It comprehended a structured script (Appendix B) for:

(i) The arousal phase, suggesting successive body awareness; 

(ii) The awareness phase, evoking consecutive and uninterrupted autobiographical memory contents from childhood to the present moment. 

In the Experimental group, the mutual aim of both arousal and awareness phases was to increase self-awareness and self-concept. In healthy individuals, this is a cognitive phenomenon associated with the well-being or *eudaemonia* [47,125]. 

On the other hand, sham intervention (b) was used as a controlled study in the Control group. Sham intervention controls memory recalling, emotion regulation, and learning in a vigil state of consciousness. It consisted of listening to the same lecture in a classroom, i.e., without any other external stimuli. The sham intervention did not comprise the intervention protocol’s focus on arousal or awareness phases. 

**Figure 6:**
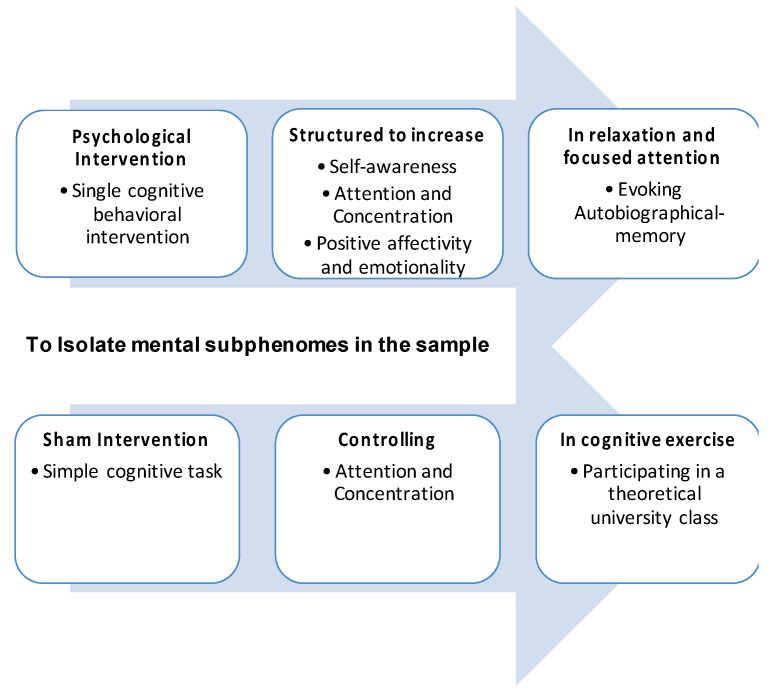
Intervention protocol, comprising the experimental procedure and the sham intervention. A diagram is presented for the graphical representation of the Intervention Protocol used to isolate the mental subphenomes in our neurotypical sample. Two horizontal axes are represented: the superior refers to the experimental procedure, and the inferior to the sham intervention. The experimental procedure, i.e., the psychological intervention, is a single cognitive behavioral intervention structured to increase self-awareness, attention, and concentration, as well as positive affectivity and emotionality. The intervention comprehends a focused evoking of autobiographical memory in a relaxed setting. The sham intervention is a simple cognitive task controlling for attention and concentration in a mental exercise of participating in a theoretical university class.

Figure 6 presents a diagram summarizing the Intervention Protocol (experimental procedure and sham intervention) and helps the reader fully understand the protocol. The outcome of the Intervention Protocol is, hence, generating different subphenomes or subtypes of subjective experience in neurotypical subjects. Those two types of intervention generated distinct cognitive functioning. 

The flow chart in Figure 4 systematizes the self-constructs, as experienced in trait mindfulness [37,38]. This Intervention Protocol aroused those constructs as a process for a neurophenomenological analysis, stressing consequently the importance of gathering first-person data as a heuristic strategy for describing and quantifying physiological processes relevant to consciousness [126]. Finally, for a detailed description of the protocol, i.e., “how it was”, “what was done”, “how was it done?” and “what was intended to compare?” adequate information is available in the author’s previous work on this subject [16,49,55]. 

### 2.5. Statistical Analysis

As in Hipólito and Martins [49], frequency tables (simple/double entries) and boxplots were used as analytical/graphic tools for the descriptive and exploratory analysis. Scale variables were summarized as mean, standard deviation, median, quartiles, minimum, maximum, and/or other order statistics when the sample distribution was justified. Categorical variables were summarized using frequency and percentages. When the scale of the measure was ordinal, order statistics were considered, e.g., tercile. Moreover, Spearman’s rank correlation coefficient was applied. For the biometric data, T-student for two independent samples and T-student for two paired samples was used to compare medians. One-way ANOVA, parametric and non-parametric, was used when comparing more than two means, i.e., the mean difference of the four subgroups in the study.

The sample size study is supported by the previous preliminary results of the Experimental group [55]. A potency (T-Student) of 80%, a significance level of 5%, and an effect size corresponding to 40% of the estimated value for the standard deviation of the variation of the molecular weights of the protein profile in the preliminary study were considered. The protein profile is defined as the detection of the character and quantity of specific sets of proteins in blood or other specimens. Protein profiling has been used to diagnose illnesses that release specific protein patterns [127,128]. Considering this option, it corresponds to a medium Cohen’s effect size; an appropriate sample size of 64 elements for each group (Experimental and Control) was found. 

#### 2.5.1. Limitations of the Psychological Assessment and Reliability Analysis

The reliability, temporal consistency of the psychological measures, and the test and retest stability were considered in the protocol elaboration and the methodology used, as well as in the definition of the objectives and the expected results. This is a descriptive study, where the intention is to observe the correlation between cognitive and physiological variables, and not a causal analysis or clinical trial, where the purpose is to objectify the effect of an intervention. It is intended, therefore, to determine the relationship between a set of psychological measures and a profile of biological markers.

Regarding the test-retest reliability of the psychological measures or repeatability, it should be noted that being a descriptive study, the priority objective is not to show causality between psychological measures, the Intervention Protocol, and molecular measures. Indeed, a mental state is in permanent change and, above all, the “state” measures. To guarantee the stability of the data, i.e., the reliability and temporal consistency of the psychological measures obtained by the standardized and structured assessment of the mental states and traits, two types of questionnaires were used: (i) those that measured states and (ii) those that measured traits. 

In the author’s previous publications [16,49], the instruments used are already analyzed, considering the problematic stability of mental states in healthy subjects. In a sequential publication, the reliability of all questionnaires in their original publication and their application to this sample will be analyzed. It should be noted that the questionnaires for measuring a state and a trait were used and validated in the Portuguese population, for that same mental state or trait, by the authors in their original publications in the Portuguese population: APZ [84], SHS [85], MAAS [86] and SCS-R [87]. 

The scales were then applied in this study with the author’s consent concerning and respecting the internal consistency of the original publication. Furthermore, the instruments used in the standardized and structured clinical interview are fully detailed in Appendix A. Case Report Form. Considering the reliability tests, internal consistency measures obtained by statistical tools such as Cronbach’s α are usually intended to study the capacity for sample separation through the instrument used. The specific Cronbach’s α was reviewed and will be published in a sequential publication, demonstrating that the four scales used to present an adequate internal consistency in the sample of neurotypical young adults compared with the internal consistency if applied to the Portuguese population. 

Given this, the reliability and internal consistency measures are conveniently calculated in a sequential publication and proposed as the test-retest reliability. However, the collection of psychological measures was done in a single time, and the study design did not include further exploration of test-retest reliability. 

#### 2.5.2. Type I and Type II Errors

We want to warn you about the limitations of conducting such an analysis and hypothesis testing. To prevent type I and type II errors, we used: (i) instrumental variables, helping to expose “hidden” variables other than the independent variables that could cause our results; (ii) purposive sampling, selecting our sample based on the knowledge about our statistical population; (iii) randomization tests, considering all possible ways to assign experimental values to all groups; and (iv) sequential assignment, assigning the first subject to the Experimental group, the second subject to the Control group, the third subjects to the Experimental group, the fourth subject to the Control group, and so forth.

### 2.6. Software 

The following pieces of software were used:

(i) IBM SPSS Statistics 22 (with a significance level = 5%) for statistical and data management;
(ii) LabWare™ LIMS and Microsoft Excel for laboratory information management and the Neurotypical DB generation.

## 3. Results

The results comprehend the data acquired in (1.) the Verbal Protocol of the Experimental Procedure and (2.) the Mental Health Profiling.

The (1.) Verbal Protocol of the Experimental Procedure is the intervention protocol used in the 128 healthy volunteers. 

The (2.) Mental Health Profiling is the strategy to characterize the mental health of the 128 neurotypical subjects.

### 3.1. Verbal Protocol of the Experimental Procedure

The Verbal Protocol (Appendix B) of the single cognitive behavioral intervention is based on the publications of Simões and colleagues [21,44,116] and was initially tested in the pilot study of this experiment [55] and as the experimental procedure of this work. The Verbal Protocol comprised a script that was kept rigorously in all study subjects, (i) to avoid confounding variables specific to the experimental procedure and (ii) to maintain the repeatability of the intervention and the reliability of the psychological measures. 

The 1st phase, the *Arousal Phase* [69,74], is a conditioning procedure [119]. With a duration of 15 min, it aims to modulate arousal or the level of consciousness. 

The 2nd phase, the *Awareness Phase* [121,122], is an expectancy procedure [123,124]. With a duration of 30 min, it aims to modulate awareness or the content of consciousness. 

This paper’s Appendix B fully presents the single cognitive behavioral intervention script. 

### 3.2. Mental Health Profiling

As published in Hipólito and Martins [49], self-report measures were used in a sample of 128 neurotypical subjects. The following psychological validated scales were applied: (i) *Abnorme Psychischer Zustände* States of Consciousness (APZ) [84]; (ii) Subjective Happiness Scale (SHS) [85]; (iii) Mindful Attention Awareness Scale (MAAS) [86]; (iv) Self-consciousness Scale—Revised version (SCS-R) [87].

Considering the complex self-regulation relationships [17] found among the constructs measured by the Assessment Protocol, our expectations for this research were solely the following:

(i) To better understand self-regulatory cognitive faculties, like self-awareness;
(ii) To deliver a more comprehensive in-depth analysis of mental health functioning [48];
(iii) To transfer this analysis into the neurobiological study of neuropsychiatric disorders.

Thus, the results of this work permitted a general phenomenological evaluation of the mental functioning of neurotypical subjects. Accordingly, the study proposed the characterization of distinct profiles, or mental health subphenomes, presented by that population. 

Firstly, and in what refers to as the Intervention Protocol, we used cognitive stimulation [49] to stratify four distinct profiles, or subphenomes, in terms of the constructs of self and bodily representations. The literature defines cognitive stimulation as an intervention providing general stimulation for thinking, concentration, and memory, usually in a social setting [129]. In our experiment, specific cognitive states were induced, and specific cognitive functions were recruited, e.g., declarative memory [130]. The Control Top subgroup exercised semantic memory during the task, whereas the Experimental Top subgroup had to exercise cognitive processes associated with episodic memory. 

As an intended outcome, this cognitive stimulation generated a more focused, awake, and aware mental state [131,132,133,134]. Secondly, and concerning the Assessment Protocol, which aimed at mental health profiling, our neurotypical sample was later studied by four self-report measures. The results of this strategy permitted the adequate profiling of the sample into four different subtypes or strata. This assessment identified and characterized four discrete mental health functioning subgroups significantly and relevantly. 

**Figure 7:**
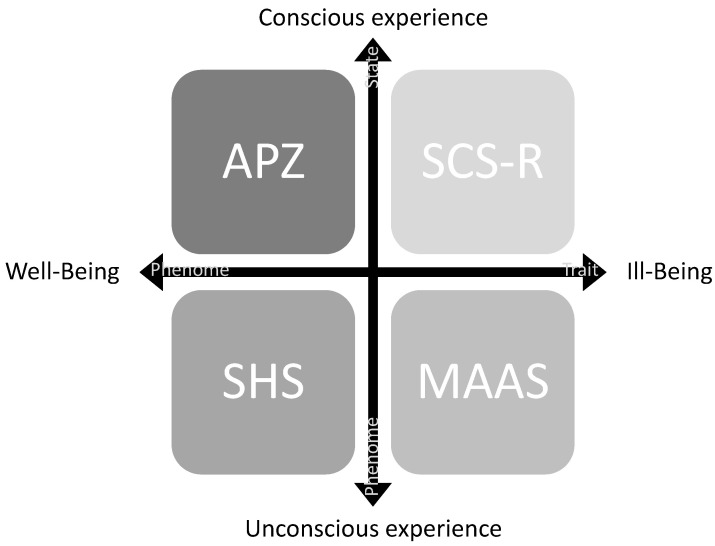
Neurophenomenological sampling and subgroup identification: Psychological Scales. A two-axis plot represents the mental health profiling and the four instruments used for the clinical stratification. The vertical axis represents the constructs of a more self-conscious experience vs. a less self-conscious experience. The horizontal axis represents the complementary constructs (postulated in this study) of a higher tendency to well-being vs. a lower tendency to well-being.

A hierarchical ranking assured this profiling of the neurotypical young adults of the self-report measures of the Psychological Assessment. This hierarchical ranking, which led to the mental health strata, was based on the pertinence of each psychological scale to evaluate the importance and degree of the mental health constructs we wanted to assess (Figure 7). 

The *Abnorme Psychischer Zustände* States of Consciousness (APZ) has the highest weighing since, in our neurotypical sample, it was first intended to identify a state of self-awareness. Likewise, APZ is the only psychological evaluation in our set of psychological scales of a state phenome, and, therefore, able to characterize the cognitive functions stimulated by the Intervention Protocol. A state phenome is thus a condition or status at a particular time characterized by the relative stability of its essential components or elements [135]. 

Subjective Happiness Scale (SHS) comes second in what concerns the weighing, as it was intended, in identifying neurotypical subjects that tend towards a well-being experience with self-consciousness traits. For this purpose, SHS tries to address, on the one hand, the personal and the external happiness trait; and, on the other hand, the absolute and relative happiness traits [47]. Therefore, SHS evaluates a phenome trait. 

These two scales (APZ, SHS) best-characterized constructs present in the top phenomes in both Experimental and Control groups (Figure 7) scored higher in the Assessment Protocol. 

Mindful Attention Awareness Scale (MAAS) comes third in terms of what concerns the weighing. The instrument was explicitly used to probe into the pre-reflective self-experience. This self-experience is essential for a passable state of self-awareness and a stable self-consciousness trait. Awareness, in a pre-reflective self, seems to be related to a cognitive construct associated with present experiences, which one is aware of without any further judgment or symbolization. MAAS evaluates a phenome state.

At last, the Self-consciousness Scale—Revised version was used in what concerns the weighing. The instrument [34] identifies, in our neurotypical sample, a core reflective self-experience and a core trait of cognition necessary for high-level reasoning and logic. The reflective self explores one’s past experiences, rationalizing previously processed concepts. As such, SCS-R evaluates a phenome trait. 

These two scales (MAAS; SCS-R) best characterize constructs present in the bottom phonemes in both Experimental and Control groups, which have scored lower in the Assessment Protocol (Figure 7).

Finally, after this classification, the self-report measures of the Psychological Assessment, *n =* 92 neurotypical young adults, were stratified into four subgroups. 

## 4. Discussion

The discussion of this experiment comprises the (1.) Stratification of the Neurotypical Sample and the (2.) Mental Status Examination.

### 4.1. Stratification of the Neurotypical Sample 

Through a cognitive behavioral intervention, a more conscious experience was aroused. Afterward, the participants were questioned to assess their experience. 

The participants were subsequently separated into independent groups:

(a) The experimental group (experimental procedure) (64 subjects);
(b) Control group (sham intervention) (64 subjects).

Then, these two groups were separated by a proportionate stratified random sampling into the Top and Bottom Phenomes, which will contribute to the interpretation of this pre-molecular screening. This first clinical subtyping permitted a stratification resulting in four subgroups: (i) the Experimental Top Phenome; (ii) the Control Top Phenome; (iii) the Experimental Bottom Phenome; and (iv) the Control Bottom Phenome. 

As shown in Hipólito and Martins [49], these four subphenomes (subgroups) were considered comparable for age, gender, and BMI, and there is a clear distinction between the experimental and control subgroups. 

### 4.2. Mental Status Examination 

A standardized and structured clinical interview was made using the following four psychological scales. This classical mental status examination in a sample of neurotypical subjects allowed the systematic characterization of the (1) Experimental Top subgroup, the (2) Control Top subgroup; the (3) Experimental Bottom subgroup; and the (4) Control Bottom subgroup. This psychological evaluation estimated the (1) process and content of thought and perception (APZ), (2) mood and affect (SHS), (3) cognitive awareness and attention (MAAS), and (4) insight and judgment (SCS-R) of the four healthy subgroups.

The (1) Experimental Top subgroup is defined by no alterations in the process and content of thought and with a conserved perception. The stream and rate of thoughts are adequate (quantity and tempo), as well as their flowing and rational connection or logical coherence. There is no exhibited loosening of associations or flight of ideas. Parallelly, the qualitative properties of thinking and its intensity, salience, and associated emotions are optimal. No delusions or hallucinations are present. The organization, identification, and interpretation of sensory information are preserved to correctly and accurately apprehend the experimental procedure. Henceforth, there are no negative alterations of thought or perceptual changes. Still, an intense emotional response, specific bodily schema changes, and meaning alterations are characteristically induced during cognitive behavioral interventions.

The mood is reported as “happy”, viz., the predominant and prevalent internal/subjective state at any one time. The emotional type is characterized as euphoric and associated with a deeply felt associated mood. The affect is characterized by neither a depressive nor anxious dimension. The external and dynamic manifestation of the subjects’ internal emotional state is described as intense, not labile, and congruent to the global content of the descriptions after the experimental procedure. Absolute and relative emotions of well-being are pronounced and defined as stable. Self- and non-self perception of subjective happiness is evoked. Thus, the subjects in this subgroup balance positive and negative emotions but also can make a judgment of the overall life quality. This judgment is likely not comparable to an ordinary sum of the subjects’ latest levels of affect and satisfaction with life but indicative of an overall affective and emotional perception.

Cognition and, more specifically, the level of consciousness, alertness, and attention is ideal, i.e., the awareness and responsiveness to the experimental procedure. This subgroup is characterized by the ability to maintain focus and sustain and appropriately shift mental attention. Subjects in this subgroup have a higher open awareness of the present, i.e., the exercise of immediate and short-term memory. The subjects in this subgroup show elevated private self-monitoring and cognition, targeted with the cognitive behavioral intervention. Hence, they effectively regulate emotionality and attention capacity to present internal state experiences.

The insight and judgment are highly maintained, as well as high availability and cooperation with the experiment. The subjects in this subgroup understand their mental state and are highly aware of their situation. Moreover, they can make reasoned and responsible decisions to preserve their and others’ well-being. It is characterized by participants with low impulsiveness and high social cognition and self-awareness. Thus, private and public self-consciousness is maintained and is stable over time. This mental status arises from sustained conceptual thought, long-term memory, and the capacity for abstraction and interpretation. Hereafter, they present highly developed constructs of self-reflection and self-concept and effective regulation of internal state awareness, and the ability of self-presentation and introspection.

This subgroup is thus interpreted as having a highly developed self-awareness, which is characterized by the progression from the pre-reflective self to reflective phenomenology and then to the autobiographical narrative interpretation [136,137] and high-level cognition [138]. Phenomenologically, this subgroup described the experiment as an oceanic boundlessness, i.e., experiencing a deeply felt positive mood and self-understanding. 

The (2) Control Top subgroup is characterized by no alterations in the thought process and content and a preserved perception. The stream and rate of thoughts are adequate (quantity and tempo), as well as their flowing, rational connection, logical coherence, and associations of ideas. Parallelly, the qualitative properties of thinking and its intensity, salience, and associated emotions are ideal. No delusions or hallucinations are present. The organization, identification, and interpretation of sensory information are preserved to represent correctly and accurately understand the university class’s content, i.e., the sham intervention. Henceforth, there are no negative alterations of thought or negative perceptual changes. Though there is no intense emotional response, bodily schema changes or meaning alterations are evoked during the cognitive task of attending a class, as expected.

The mood is also reported as “happy”, viz., the predominant and prevalent internal/subjective state at any one time. The emotional type is characterized as euthymic. The affect is characterized by neither a depressive nor anxious dimension. The external and dynamic manifestation of the subjects’ internal emotional state is described as mild, not labile, and congruent to the global content of the lesson. Absolute and relative emotions of well-being are designated and described as stable. Self- and non-self perception of subjective happiness is evoked. Thus, the subjects in this subgroup also balance positive and negative emotions and can judge the overall quality of life.

Cognition and, more specifically, the level of consciousness, alertness, and attention is standard, i.e., the awareness and responsiveness to focusing on the class. This subgroup is also characterized by the ability to maintain focus, appropriately shift mental attention, and exercise immediate and short-term memory.

The insight and judgment are maintained, as well as the interest in the contents of the class, i.e., the sham intervention. The subjects in this subgroup understand their mental state and situation and have high social cognition. Moreover, they can make reasoned and responsible decisions to preserve their and others’ well-being. Thus, private and public self-consciousness is maintained and is relatively stable over time. Likewise, they present highly developed constructs of self-reflection and self-concept, effective regulation of internal state awareness, and the capacity for self-presentation and introspection.

This subgroup is interpreted as having a high self-consciousness, which can be characterized as having pre-reflective bodily sensations and emotional states in a 1st or 2nd person interaction with the environment, which can be described as being-in-the-world or a natural attitude [14,63,139,140]. 

The (3) Experimental Bottom subgroup is also characterized by no alterations in the thought process and content and with a still preserved perception. The train of thought is held, but the rate of negative thoughts is slightly increased (quantity and tempo), somewhat affecting the flowing and rational connection or logical coherence. Still, there is no pathological loosening of associations or flight of ideas. The qualitative properties of thinking and the associated emotions are less congruent. Hence, the organization and interpretation of sensory information are less preserved and somewhat affect the experimental procedure’s adequate representation and understanding. Still, no delusions or hallucinations are present. Henceforth, there are no negative alterations of thought or perceptual changes, nor an emotional response evoked during the cognitive behavioral intervention.

The mood is less reported as “happy”. The predominant and prevalent internal/subjective state is characterized as slightly apathetic. The affect is not pathological, but intermittent and has a minor anxious dimension. The external and dynamic manifestation of the subjects’ internal emotional state is described as marginally labile and somewhat not utterly congruent with the personal descriptions after the experimental procedure. Scarce absolute and relative emotions of well-being are expressed, and limited self-perception of subjective happiness is evoked. 

The level of consciousness, alertness, and attention is ordinary, i.e., the awareness and responsiveness to the experimental procedure. This subgroup is characterized by some tendency to inattention and slight difficulties sustaining concentration. Subjects in this subgroup have a lower open awareness of the present. 

The insight and judgment are maintained, but the subjects in this subgroup understand less their mental state and have a lower level of awareness of their situation. Participants with some propensity to impulsiveness characterize it, showing lower social cognition and self-awareness. Private and public self-consciousness is maintained but somewhat unstable. 

This subgroup is interpreted as having a highly reflective self-experience. Reflective self-experience is a procedural cognitive process that occurs whenever “one inspects, or reflectively introspects one’s experience, or recognizes one’s specular image in the mirror, or refers to oneself with the use of the first-person pronoun, or constructs a self-narrative” [141]. Therefore, this precedes the content to which self-focus is addressed. In other words, it is a reflective experience intentionally directed toward conscious thought. 

The (4) Control Bottom subgroup is characterized by no alterations in the thought process and content and a preserved perception. There is no pathological loosening of associations or flight of ideas, but the qualitative properties of thinking and the associated emotions are less congruent. Hence, the organization and interpretation of sensory information are less preserved, affecting the adequate representation and understanding of the contents of the university lesson, i.e., the sham intervention. However, as in the other subgroups, no delusions or hallucinations exist. Henceforth, there are no negative alterations of thought or perceptual changes, nor an emotional response evoked during the cognitive task of attending a class.

The mood is less reported as “happy”. The predominant and prevalent internal/subjective state is characterized as dysphoric. The affect is defined by not pathological but intermittent and minor depressive dimensions. The external and dynamic manifestation of the subjects’ internal emotional state is described as slightly labile and somewhat not wholly congruent with the global content of the lesson. Few absolute and relative emotions of well-being are expressed, and reduced distinctive self-perception of subjective happiness is evoked. 

The level of consciousness, alertness, and attention is average, i.e., the awareness and responsiveness to focusing on the class. Some propensity also characterizes this subgroup to inattention and slight difficulties sustaining concentration. Likewise, subjects in this subgroup have a lower open awareness of the present. 

The insight and judgment are kept, but the subjects in this subgroup understand their mental state and have a lower awareness of their situation. It is characterized by participants with some predisposition to impulsiveness, presenting a lower social cognition and self-awareness. Private and public self-consciousness is maintained but slightly less stable. 

This subgroup is interpreted as having a pre-reflective self-experience, embodied and situated cognition. Pre-reflective is (1) an awareness occurring before and reflecting on the experience; (2) it is implicit. “An explicit reflective self-consciousness is possible only because there is a pre-reflective self-awareness that is an ongoing and primary self-consciousness. In other words, the experiential dimension always involves a significant implicit pre-reflective self-awareness” [141]. In agreement with Husserl [139], the “notion of pre-reflective self-awareness is the idea that experiences have a subjective ‘feel’, a certain phenomenal quality of ‘what is like’ (a perceptual experience) or ‘what it ‘feels’ like to have them (bodily sensation)”. 

Such “first-person experience presents us with an immediate and non-observational access to ourselves in a (minimal) form of self-consciousness” [141].

**Figure 8:**
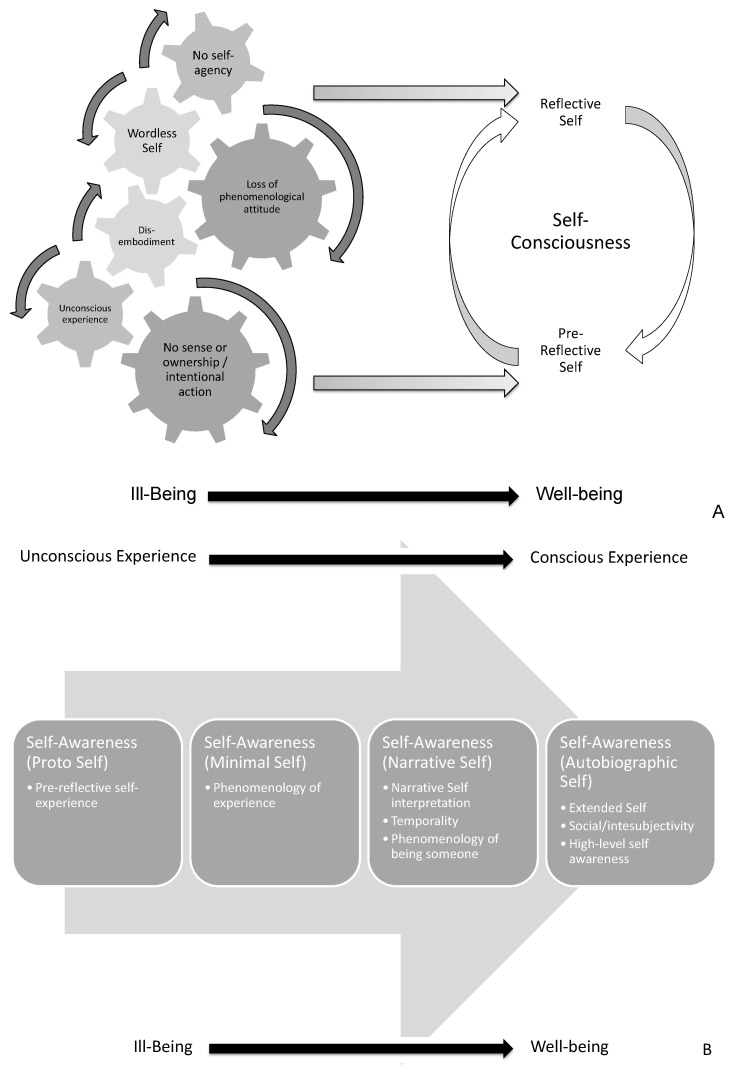
Sample characterization and neurophenomenology schematics (**A**,**B**). 2 graphical representations of the cognition of the neurotypical self are presented. In (**A**), the different constructs of self-consciousness are introduced. In (**B**), self-awareness is shown as a progression in a 2-dimensional axis: (a) unconscious to conscious experience and (b) ill-being to well-being.

From an epistemological perspective, there are two different epistemological self-experiences to be distinguished: pre-reflective and reflective [141]. The dynamics between pre-reflective and reflective experience and the progression to self-conscious/aware experiences may result in a healthy self-experience. This dynamic will be further explored in sequential publications and is summarized in Figure 8A.

Thus, this dynamic is exposed in cognition of the neurotypical self and chronic pathologies [142,143,144] or neuropsychiatric syndromes, e.g., schizophrenia and autism [145]. Ultimately, self-awareness can be considered a progression (Figure 8B) in a 2-dimensional axis: (a) unconscious to conscious experience and (b) ill-being to well-being [47]. This progression will also be further explored in sequential publications. 

**Figure 9:**
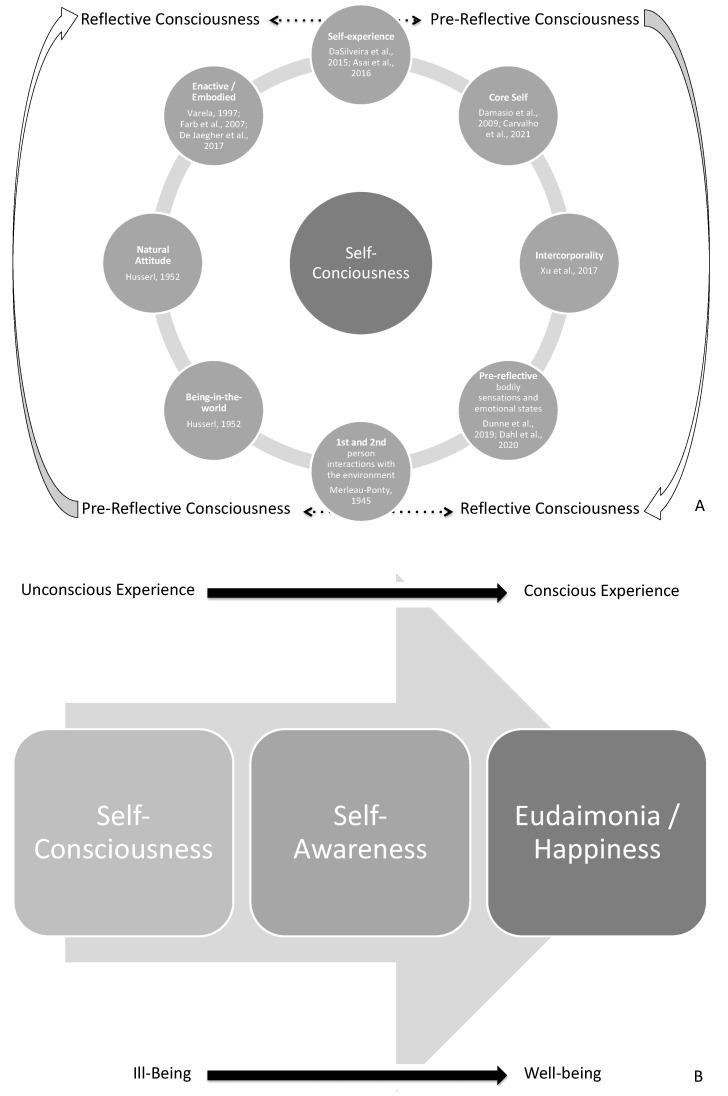
Sample characterization and neurophenomenology schematics (**A**,**B**). 2 graphical representations of the cognition of the neurotypical self are presented. In (**A**), the self-consciousness trait is shown as a prerequisite to self-awareness. The self-consciousness constructs examined in the paper are schemed: self-experience [34,35], core self [117,120], intercorporality [114], pre-reflective bodily sensations and emotional states [14,63], 1st and 2nd person interactions with the environment [140], being-in-the-world [139], natural attitude [139] and enactive/embodied [29,59,136]. In (**B**), it is given a conceptual analysis of well-being.

As a synthesis of our introduction and obtained results, the self-consciousness trait is a prerequisite to that progression (Figure 9A). It is postulated that that progression may result from well-being or eudaemonia in neurotypical subjects (Figure 9B).

**Figure 10:**
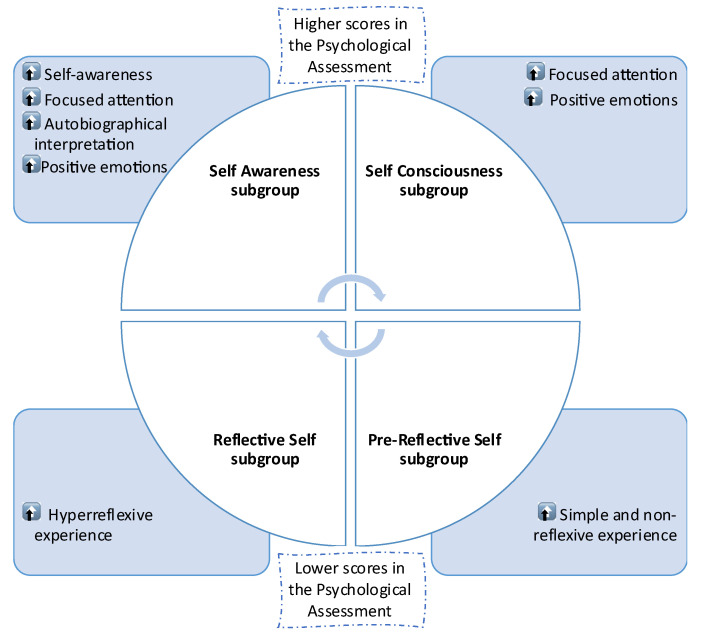
Mental health subphenomes in a neurotypical sample. A diagram is presented for the characterization of 4 the mental subphenomes isolated by the Psychological Assessment: i. the Self Awareness subgroup; ii. the Self Consciousness subgroup; iii. the Reflective Self subgroup, and the iv. the Pre-Reflective Self subgroup. The neurotypical sample is divided into four functional subgroups depending on the psychological assessment scores. A brief description of the mental status of each functional subgroup is presented.

Finally, as presented in Figure 10 and resulting from our previous analysis, the:

(1) Top phenome of the Experimental group is defined as the “Self Awareness subgroup”;
(2) Top phenome of the Control group is defined as the “Self Consciousness subgroup”;
(3) Bottom phenome of the Experimental group is defined as the “Reflective Self subgroup”;
(4) Bottom phenome of the Control group is defined as the “Pre-Reflective Self subgroup”.

## 5. Conclusions

A clinical or pre-molecular screening necessary for efficient molecular biomarker research of neurotypical subjects was proposed. With this study, we primarily aimed to find distinct mental health profiles of individuals without a neuropsychiatric condition. 

It is known that in neurobiological functional response studies [146], i.e., in explanatory research, profiles of cognitive functioning, like those found in this study, may show a homogeneous molecular response. 

This functional response has been mainly documented in the neurotypical individual [147,148]. Therefore, this paper attempted to present the possible inference between cognitive and biological substrates, which the authors will then test. 

Initially, the intervention and assessment protocols stratified 128 neurotypical young adults by a standardized and structured interview using the following scales APZ, SHS, MAAS, and the SCS-R. This evaluation permitted thus the subdivision into four subgroups. 

First, the Self Awareness subgroup, which is a mental subphenome characterized not only by an increased self-awareness with focused attention but also by an augmented autobiographical interpretation with the expression of positive emotions.

Second, the Self Consciousness subgroup, which is a mental subphenome characterized by focused attention and the expression of positive emotions.

Third, the Reflective Self subgroup, which is a mental subphenome characterized by a hyper-reflexive experience.

Forth, the Pre-Reflective Self subgroup, which is a mental subphenome characterized by a simple and non-reflexive experience.

Lastly, this analysis permitted the isolation of four mental health strata, or subphenomes, with *n =* 23 each. These four subphenomes present different cognitive characteristics, which might also have distinct molecular expressions. Hence, each of those four mental health subphenomes (totalizing *n =* 92 neurotypical young adults) may have a distinct neurophysiology, a discrete molecular fingerprint, and a variate likelihood of developing a neurological or mental condition. 

**Figure 11:**
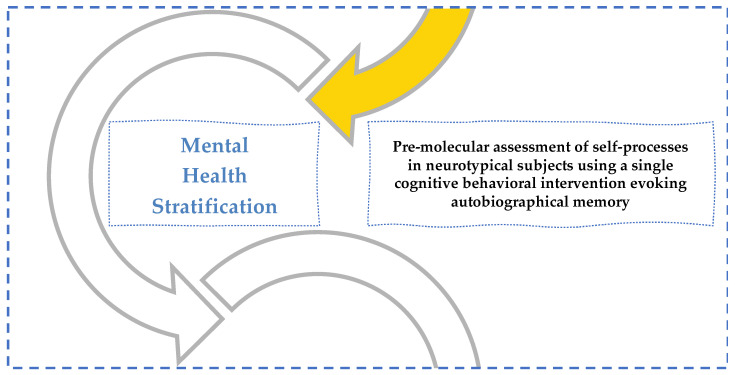
Research Outline: Mental Health Stratification Graphical scheme presenting the integration of this paper in the overall experiment conducted by the authors for mental health stratification of a neurotypical sample. “Pre-molecular assessment of self-processes in neurotypical subjects using a single cognitive behavioral intervention evoking autobiographical memory” comprehends thus the intervention and assessment protocols used in 128 healthy university students.

In conclusion, the following research outline (Figure 11) helps the reader understand this paper’s sequence and integration into the overall experiment conducted by the authors for the mental health stratification of 128 healthy university students.

### Outcomes and Limitations

In order not to exceed the findings and to be consistent with the objectives, the conclusions of this paper are presented exclusively about the convenience sample. The observed results point only to a simple categorization of mental health states and traits to avoid clear errors in the statistical generalization process. Consequently, this type of descriptive research must be validated by explanatory research, i.e., a follow-up causalistic study with a higher sample size and internal validity. This descriptive research is, to a certain degree, a proof of concept, providing preliminary explanatory assessments and correlations between self-processes.

In detail, a complete a priori mental health stratification is executed for adequate biomolecular research of a sample of 128 neurotypical subjects. To better separate and isolate cognitive strata in neurotypical subjects, an experimental procedure consisting of a psychological experiment based on a cognitive behavioral intervention was created. The result was the screening of 4 distinct mental health profiles and an empirical correlation between complex self-processes.

The mental status of neurotypical subjects is not yet thoroughly investigated for what was recently published. Hence, this strategy characterized and stratified a neurotypical sample into four discrete subgroups and explained how self-awareness representations (bodily, autobiography, proto, and narrative self) would serve as pre-molecular typing. 

In this experiment, one of the objectives of our experimental procedure, composed of two phases (arousal and awareness phases), was to increase positive mood, especially in the awareness phase. The sham intervention controlled mainly for attention and concentration; thus, the positive mood did influence the results. However, the distinct self-concept representations present in the four obtained profiles have included positive mood as an evaluated variable. We consider that positive mood promotes bodily awareness, principally if combined with the progressive relaxation used in the arousal phase of the experimental procedure. As an alternative to our protocol, we also believe that progressive body-scan techniques may be a reasonable procedure that does not manipulate relaxation while focusing directly on bodily awareness. 

Moreover, and to include adequate molecular descriptions to complement this psychological analysis, the acquisition of proteomics data will be provided in further publications of this experiment. With the data obtained in this paper proving to be a promising framework and research design, a physiological approach was thus prepared to assess protein network changes fully.

Henceforth, in a sequential publication, brain protein information by an in silico analysis is predicted [149]. Later, the total protein profile characterized by capillary electrophoresis is foreseen [55]. Finally, it is projected to assess neurobiological processes in subjects without neuropsychiatric conditions by simultaneous immunodetection and quantification of their neuropeptide and neuroimmune response.

Concerning the pre-and post-treatment comparison of the scores obtained in the whole experiment, we calculated the difference [T1; T0] for the molecular analysis. The pre-and post-treatment comparison was not estimated for the psychological analysis, as it was not intended to study the effect of the experimental protocol on the variables obtained on the self-report measures, i.e., participants being measured on APZ, SCS-R, SHS, and MAAS before and after the interventions. 

Indeed, for the psychological analysis obtained by the self-report measures, what was intended was to stratify and identify subphenomes of psychological functioning, as this study was not a clinical trial to address the efficacy of an experimental procedure but rather a descriptive type of study.

As referred before, the reliability of the cognitive states and traits will be tested by the measures used in a future publication. The mental status of the participants is maintained over time. The mental state’s stability remains similar in the absence of the cognitive behavioral intervention and in the sham intervention used in this experiment since it is a fundamental nature of a mental state if no external stimulation is given. In the experimental procedure, because there was only a single cognitive behavioral intervention [27], the modification (or “instability”) of the psychological measures is to be attributed to this intervention.

Again, note that the psychological variable studied is traces of autobiographical memory, which is a measure that remains constant in neurotypical individuals. Therefore, the stability of self-conscious experience is maintained because there were no external stimuli not adequately controlled or monitored. 

As the main conclusion, the problem with such research is that self-conscious experience in a neurotypical population varies with time and external stimuli if not adequately controlled. The question is whether there are cognitive and physiologic states and traits that maintain continuity and remain stable over time.

## Data Availability

This study uses a publicly available Case Report Form (CRF) and Verbal Protocol. The CRF comprises the instruments used in the standardized and structured clinical interview, showing the items evaluated on each psychological scale and the questions posed to the participants for each self-report measure. This data can be found in Appendix A [150], https://doi.org/10.5281/zenodo.6945708. The Verbal Protocol is based on the publications of Simões and colleagues [21,44,116]. This protocol was initially tested in the pilot study of this experiment [16]. The Verbal Protocol is used in the single cognitive behavioral intervention as a script and comprises the 1st phase, the *Arousal Phase*, and the 2nd phase, the *Awareness Phase*. This data can be found in Appendix B [151], https://doi.org/10.5281/zenodo.7125291.

## Appendix B. Verbal Protocol

The single cognitive behavioral intervention script comprises the 1st phase, the *Arousal Phase*, and the 2nd phase, the *Awareness Phase*.

**1st Phase, The *Arousal Phase***

*Close your eyes... Feel good and quiet with your eyes closed... Offer this moment to be with you fully... In silence... In peace...*

*Breathe deeply...*

*Hold your breath for a few moments...*

*Let go of the air slowly... And relax...*

*Once again...*

*Breathe deeply, feeling comfortably pleasant... Moreover, loosen the tensions of your body...*

*Relax... Now breathe deeply... Moreover, feel comfortably safe...*

*Comfortably protected... Release the air slowly through your nose...*

*Um.*

*What a pleasurable feeling to become aware* *of the air that enters and nourishes your body.*

*Your body is all nourished by this clear, comfortably healthy air that oxygenates your body and emotions.*

*Feel your body now.*

*Imagine a light, your favorite color...*

*Wrapped by a cloud in the form of soft cotton filling your body.*

*Um! Feel this colorful cloud filling your feet.*

*Become aware of your left foot... Relax it.*

*Become aware of your right foot... Relax it.*

*Now let this soft, colorful cloud fill your legs...*

*Relax your left leg.*

*Relax your right leg...*

*This feeling of relaxation also continues by your belly... Relax your abdomen...*

*Relax your heart...*

*Relax your arms... Hands... Fingers...*

*Breathe once again deeply...*

*Feel your body relaxed...*

*Relax your posterior region; your back feels filled with this colorful cloud...*

*Your body is relaxed, relaxed.*

*Take it, take it, light.*

*How nice to enjoy being light?*

*Moreover, this colorful cloud continues to increase...*

*Gently filling your face.*

*Unwinding all your face.*

*Your face, your mouth... Relax your tongue inside your mouth... Relax your eyes... Relax your forehead... Your nose...*

*The air comes in with great fluidity now...*

*Relax your ears...*

*Um! What a nice feeling to relax all your hair...*

*Gently filled by this little colorful cloud, touching all your head...*

*Um... That feels comfortably healthy*

*Comfortably nice...*

*Comfortably in peace...*

*In peace.*

*(pause)*

**2nd phase, the *awareness phase***

*Lightness, delicacy... Well-being is with you now...*

*You feel so light, so light, so soft that you are...*

*In pleasant situations of your childhood.*

*It could be a day with your old friends at school... Or who knows... Your neighbors...*

*A great joke that* *amused you and made him smile joyfully!*

*You are in the moment now.*

*Maybe a day of partying...*

*A long-awaited party, or maybe a party in your town, or a party at school...*

*You see nice, fun things going on at this event... It is so good to be there...*

*Um!*

*Furthermore, that person who was always with his family... It could be an aunt who liked it so much... Who knows, a friend of your mother’s... Or maybe a neighbor who talked about funny things or gave him incredible moments... Or some fantastic gift.*

*It is a birthday. It may have been yours or a friend’s... A birthday party...*

*Um!*

*That piece of cake! They sing congratulations... The games with the little friends... The noise of the voices of adults and children as a song...*

*The birthday table... The sweets... The cake!*

*Oh! The piece of birthday cake...*

*What a delight! Was it chocolate? Was it a fruit? What was your favorite cake? And present... What is the gift from your childhood you liked?*

*What would you like to play with and with whom?*

*What about the tv? Which cartoons would you most like to watch?*

*And the movies... And the series?*

*What about school? Do you remember when the day you started reading?*

*The pleasure of putting the little letters together and understanding what they were saying?*

*What about the break? What would you like to do at halftime?*

*What about the school snacks?*

*What about the teacher you admired the most? What did you think was beautiful at school?*

*A special poster? A special tree? A room? A corner of the school where he felt good...*

*What about your friends at school? What you talked about... I would be thrilled.*

*Moreover, what did you learn? What was the theme of the school you liked to know?*

*What about the study visits? Where was the best? What was interesting there?*

*And the games... The singing inside the tour bus... So much joy on this journey...*

*What about your favorite food?*

*Um! Remember that tasty ice cream that made you grow mouthwatering?*

*Or that sweet guy who adoptees! He savored every pleasant moment of his childhood.*

*(pause)*

*What about nature? Have you ever played with the rainwater? Or have you ever showered in rainwater? Furthermore, play with the flowers and the insects...*

*And some pet of yours or a friend... A cat... A dog... A little bird*

*Oh! Furthermore, the ladybugs, when they appreciate it near us... Butterflies...*

*Anyway... Your favorite toy when you were a kid... Moreover, his nickname made him angry, but he knew that whoever called him that was because, deep down, he loved him so much.*

*Moreover, when he was sick, someone took care of him with much pampering... Soap or a particular food that day...*

*And parties like Christmas... Did you believe in Santa Claus?*

*Did you write him letters? Was there exceptional food on Christmas day? Family reunion?*

*What about other essential parties in your childhood? Feel all these positive memories of your childhood... Be there now... Let your memories come up...*

*With images... Sounds... Body sensations... Smells...*

*All! Relating his childhood to phases of joy.*

*Allow this pleasurable feeling to pack your body and emotions now...*

*Breathe for a moment and visualize a colorful, beautiful, crisp picture representing your childhood! Your happy phase! Keep this happy childhood phase inside you...*

*And...*

*whenever you remember a scene from your childhood... In the happy phase... I will have a sense of well-being... And happiness.*

*(repeat pause three times this sentence, and whispering last time)*

*Moreover, now breathe deeply.*

*Release the air slowly...*

*You are a teenager now.*

*A teenager...*

*Look at your teenage body...*

*What was the style of that time? The kind of clothes I would like to wear...*

*If you are a man, your favorite pants, the clothes you are cutest with, your sneakers... Did you use anything else noteworthy?*

*What did you do with your physical appearance to feel better... More beautiful?*

*Moreover, if you are a woman. What did you wear that you liked very much? The necklaces, earrings, haircut, make-up, lipstick, shadow, and unique way of feeling like a teenager.*

*What about the changes in the body? Stop being a girl or a boy and feel like a man or a woman?*

*How did you feel about growing up? And the compliments... He is already a boy. A girl...*

*What about your friends? What about your class?*

*When did you start going out alone? Where were you going?*

*Are you hanging out with your colleagues? With friends?*

*Go to the movies? Watch movies on the net?*

*Games on the net?*

*Moreover, the messages sent?...*

*Furthermore, the first kiss or kiss that you missed...*

*And that special boy... Or that special girl in your life.*

*And the pleasant memories of this phase...*

*And the school? What was good?*

*New interests?*

*And your projects... Your desires...*

*And the parties with the friends of the class... Extraordinary pranks that just remembering makes you want to laugh... What you had that caught other people’s attention... Your smile... Your look... The way he spoke... Their way of dialoguing... What was his strength?... Yes, it had an extraordinary way of being... Feel that moment... This moment...*

*Furthermore, the songs I spent hours singing... The clips of your songs...*

*And your dreams awake... Thought about being admired by many people? Conquer the love of your life/ have the car of your dreams? Have a fantastic future with your way of being... Magical solutions to all problems...*

*Savor and intensify your imagination in everything that relates to your youth in happy moments... Generally, they are significant situations for you... Furthermore, that is nice to remember at this time... Get involved in your happy youth phase... See the colors of this phase... The smell... The special people for themselves, the sounds of this era... Experience.*

*Remember this phase of happy youth now...*

*Moreover, know that every time you remember a positive memory of your happy youth... Immediately, a sense of well-being will occur... At any time in his life.*

*(repeat pause three times this sentence, and whispering last time)*

*Breathe deeply and allow this phase of your happy youth to be within you*.

*(pause)*

*Breathe deeply again... Feel that you are in a new phase of your life.*

*It is an adult being.*

*Go to positive memories of this phase of your life...*

*Look at the dreams you have managed to fulfill...*

*Study to a certain level... The effort this has caused you...*

*Take responsibility... Yes, because being an adult is to walk with its objectives with independence and determination.*

*See your skills and skills... What is suitable to do... A drawing, a painting, a dissertation, poetry, writing...*

*Your significant achievements in your life...*

*The conclusion of the bachelor’s degree...*

*What most positively marked this stage of completion of studies...*

*Your marriage or marriage with someone who believed it could make you happier... Your professional side... His accomplishments in the work that made him happy...*

*A compliment was received... An adult achievement...*

*A walk... A movie... A book... A scene... The birth of a future son... The acquisition of an object after much idealizes a car or setting up a house... A project carried out.*

*Real friends... Which makes you feel happy...*

*The encounter with the family...*

*A day of well-being with friends...*

*A party... A diversion...*

*A wake-up after a deeply well-slept night...*

*The savor of life... A sunset... A walk... A dance... Listen to a particular song... Feel at peace!*

*See a great friend... The motivation of a special meeting with someone affectionately special to him...*

*Being entire with you at any given time... The flow with you...*

*Alternatively, if you love... To be pleased with your own company... To learn something new... The curiosity of life...*

*Being in a relaxing place... It can be on a beach, a field, a mountain, or perhaps in a place created only by you.*

*Savor this place...*

*Feel... The smell of clean clothes... Wet earth smell... The smell of a perfume that is special to him... The scent of the morning in the spring... The fragrance of your favorite flower...*

*Moreover, colors that make you sound... Feel your blue... Or it is yellow... Who knows the red... Feel your favorite color and the well-being that it provides you... Alternatively, see the objects you admire... A painting, a poem, a photograph, a city... Images that provide your well-being...*

*And your favorite songs... The sound... The melody... The lyrics and musicality...*

*Your relationship with nature... The time well lived in contact with nature... A beach, a lake, a mountain... A garden...*

*A moment when you felt that his duty was fulfilled... Managed to finish a task or project that wished very much... Something simple, like riding a mobile with the instructions or making a recipe for a cake that worked... Or even something more elaborate like organizing a meeting, learning a language... Computer science... The welfare of overcoming obstacles.*

*Anyway, rescue your positive memories from being an adult.*

*Give yourself at this moment with all the visual, synesthetic, auditory, and taste... Entirely to the pleasure of being an adult...*

*Furthermore, immediately, a sense of pleasure and well-being will come to you every time, a reminder of your phase of being a happy adult.*

*(repeat pause three times this sentence, and whispering last time)*

*Breathe deeply...*

*Now he is a person who already has a good life trajectory.*

*You have experienced many things in life.*

*What have you learned positively in the latter?*

*What did you accomplish in life that gave you pleasure?*

*How can you enjoy your time with greater creativity?*

*Have you learned it?*

*What positive challenges do you still want to achieve?*

*Determine that from now on, you will be a more positive person in life.*

*What do you want to leave as a landmark here on earth with your experience?*

*Which people let your heart smile?*

*What makes you feel good?*

*See your life project for everyday life.*

*Let it be simple, but that it means meaning to you.*

*Feel happy that life gives you the happiness of being alive.*

*Feel light and happy to have continued your trajectory so far... That will continue.*

*Breathe deeply and experience fully and healthily this phase of maturity in which you are.*

*And... Know that every time you remember this current phase of yours, your positive memories of your wisdom will provide you with serenity and well-being.*

*Live it deeply...*

*Breathe deeply... Let this well-being inside you.*

*And... Once again, breathe deeply...*

*Create a picture where you can be represented with positive memories of your childhood, adolescence, and adulthood...*

*This painting is your production... He is precious... It is a unique example in the world... It Is your positive life trajectory.*

*This picture has a pleasant smell, sounds that reach your ears well, images that delight your vision, and a texture that makes you suitable for the body.*

*Place this painting in a place that is very special to you.*

*Now give your painting a name. And remember... Every time you visit this picture of your positive memories, a pleasant sense of well-being, peace, serenity, and stillness will involve your whole being.*

*View your frame again.*

*From your positive memories... Moreover, feel the lightness of being, in peace and serene...*

*Breathe deeply*

*(pause)*

*Once again, visit your positive memories framework... Moreover, feel comfortably happy.*

*Breathe deeply...*

*(pause)*

*The time has come to return to the here and now...*

*I am going to count from 1 to 3...*

*Moreover, by reaching three, you will open your eyes with a sense of peace, harmony, and happiness.*

*Furthermore, every time the positive memories of your life trajectory appear, you will feel comfortably happy, at peace, and healthy.*

*1. Become aware of your breath.*

*2. Tome awareness of your body. Move your feet, your hands, your head. Take a vague understanding of my voice... Of the noises around...*

*3. Soft and slowly open your eyes, squirt the whole body... Stretch... Feeling here and now, in this room, in the city... Here and now! Having in itself the feeling of well-being and happiness!*

## References

[1] Trent R.J. (2005). Molecular Medicine: Genomics to Personalized Healthcare.

[2] Lawrence E. (2005). Henderson’s Dictionary of Biology.

[3] Stamos D.N. (2003). The Species Problem: Biological Species, Ontology, and the Metaphysics of Biology.

[4] Andreassen S.N., Ben Ezra M., Scheibye-Knudsen M. (2019). A defined human aging phenome. Aging.

[5] Cheng K.C., Katz S.R., Lin A.Y., Xin X., Ding Y. (2016). Whole-Organism cellular pathology: A systems approach to phenomics. Adv. Genet..

[6] Lewontin R. (2011). The Genotype/Phenotype Distinction. Stanford Encyclopedia of Philosophy.

[7] Shafer A.T. (2015). Neural Correlates of Emotion-Cognition Interactions in Healthy Functioning and Adolescent Psychopathology. Doctoral Dissertation.

[8] Siebner H.R., Callicott J.H., Sommer T., Mattay V.S. (2009). From the genome to the phenome and back: Linking genes with human brain function and structure using genetically informed neuroimaging. Neuroscience.

[9] Williams D., Schmitt M., Wheeler Q. (2016). The Future of Phylogenetic Systematics: The Legacy of Willi Hennig.

[10] Austin J.H. (1998). Zen and the brain: Toward an understanding of meditation and consciousness. Camb. Mass. Inst. Technol..

[11] Shapiro D.H., Walsh R.N. (1984). Meditation: Classic and Contemporary Perspectives.

[12] West M.A. (2015). The Psychology of Meditation: Research and Practice.

[13] Lutz A., Slagter H.A., Dunne J.D., Davidson R.J. (2008). Attention regulation and monitoring in meditation. Trends Cogn. Sci..

[14] Dahl C.J., Wilson-Mendenhall C.D., Davidson R.J. (2020). The plasticity of well-being: A training-based framework for the cultivation of human flourishing. Proc. Natl. Acad. Sci. USA.

[15] Perszyk D. (2013). Neurotypical. Encyclopedia of Autism Spectrum Disorders.

[16] Martins J.E., Simões M., Rosa N., D’Alimonte D., Mendes V.M., Correia M.J., Barros M., Manadas B. (2016). Happiness as a self state and trait of consciousness: Saliva molecular biomarkers—A brief revision. Exp. Pathol. Health Sci. Res. Clin. Teach. Soc..

[17] Carver C.S., Scheier M.F., Ryan R.M. (2019). A self-regulatory viewpoint on human behavior. The Oxford Handbook of Human Motivation.

[18] Popova T., Kourova O., Korykalov Y., Kokoreva E., Maksutova G. (2019). Psychophysical Self-Regulation Training Is Prerequisite for Human Psychophysical Safety. 2019 International Conference on Pedagogy, Communication and Sociology (ICPCS 2019).

[19] Cahn B.R., Polich J. (2009). Meditation (Vipassana) and the P3a event-related brain potential. Int. J. Psychophysiol..

[20] Cahn B. (2022). P244. Enhanced Frontal Midline Theta (fmTheta) Power after Mindfulness Training in Major Depressive Disorder (MDD) Patients Correlates With Improvements in Mindfulness, Depression, and Perceived Stress. Biol. Psychiatry.

[21] Simões M. (2002). Altered States of Consciousness and Psychotherapy. Int. J..

[22] Baron R.A., Byrne D., Branscombe N.R. (2006). Social Psychology.

[23] Cahn B.R., Polich J. (2006). Meditation states and traits: EEG, ERP, and neuroimaging studies. Psychol. Bull..

[24] Sobrinho L.G., Simões M., Barbosa L., Raposo J.F., Pratas S., Fernandes P.L., Santos M.A. (2003). Cortisol, prolactin, growth hormone and neurovegetative responses to emotions elicited during an hypnoidal state. Psychoneuroendocrinology.

[25] Hagerty M.R., Isaacs J., Brasington L., Shupe L., Fetz E.E., Cramer S.C. (2013). The case study of ecstatic meditation is fMRI and EEG evidence of self-stimulating a reward system. Neural Plast..

[26] Hanson R., Shapiro S., Hutton-Thamm E., Hagerty M.R., Sullivan K.P. (2021). Learning to learn from positive experiences. J. Posit. Psychol..

[27] Segal Z.V., Teasdale J. (2018). Mindfulness-Based Cognitive Therapy for Depression.

[28] Kabat-Zinn J. (2003). Mindfulness-based interventions in context: Past, present, and future. Clin. Psychol. Sci. Pract..

[29] Farb N.A., Segal Z.V., Mayberg H., Bean J., McKeon D., Fatima Z., Anderson A.K. (2007). Attending to the present: Mindfulness meditation reveals distinct neural modes of self-reference. Soc. Cogn. Affect. Neurosci..

[30] Kabat-Zinn J. (2019). Foreword: Seeds of a necessary global renaissance in the making: The refining of psychology’s understanding of the nature of mind, self, and embodiment through the lens of mindfulness and its origins at a key inflection point for the species. Curr Opin Psychol..

[31] Zhang Z., Luh W.M., Duan W., Zhou G.D., Weinschenk G., Anderson A.K., Dai W. (2021). Longitudinal effects of meditation on brain resting-state functional connectivity. Sci. Rep..

[32] Bishop S.R., Lau M., Shapiro S., Carlson L., Anderson N.D., Carmody J., Segal Z.V., Abbey S., Speca M., Velting D. (2004). Mindfulness: A proposed operational definition. Clin. Psychol. Sci. Pract..

[33] Hanley A.W., Garland E.L. (2014). Dispositional mindfulness co-varies with self-reported positive reappraisal. Personal. Individ. Differ..

[34] DaSilveira A., DeSouza M.L., Gomes W.B. (2015). Self-consciousness concept and assessment in self-report measures. Front. Psychol..

[35] Asai T., Kanayama N., Imaizumi S., Koyama S., Kaganoi S. (2016). Development of Embodied Sense of Self Scale (ESSS): Exploring Everyday Experiences Induced by Anomalous Self-Representation. Front. Psychol..

[36] Garland E.L., Howard M.O. (2018). Mindfulness-based treatment of addiction: Current state of the field and envisioning the next wave of research. Addict. Sci. Clin. Pract..

[37] Brown K.W., Ryan R.M. (2003). The benefits of being present: Mindfulness and its role in psychological well-being. J. Personal. Soc. Psychol..

[38] Ludwig V.U., Brown K.W., Brewer J.A. (2020). Self-regulation without force: Can awareness leverage reward to drive behavior change?. Perspect. Psychol. Sci..

[39] Aftanas L., Golosheykin S. (2005). Impact of regular meditation practice on EEG activity at rest and during evoked negative emotions. Int. J. Neurosci..

[40] Cardaciotto L., Herbert J.D., Forman E.M., Moitra E., Farrow V. (2008). The assessment of present-moment awareness and acceptance the Philadelphia mindfulness scale. Assessment.

[41] Morgan M.C., Cardaciotto L., Moon S., Marks D. (2020). Validation of the Philadelphia Mindfulness Scale on experienced meditators and nonmeditators. J. Clin. Psychol..

[42] Halsband U., Mueller S., Hinterberger T., Strickner S. (2009). Plasticity changes in the brain in hypnosis and meditation. Contemp. Hypn..

[43] Hinterberger T., Kamei T., Walach H. (2011). Psychophysiological classification and staging of mental states during meditative practice. Biomed. Tech. Biomed. Eng..

[44] Simões M., Barbosa L., Gonçalves S., Pimentel T., Fernandes P., Correia J., Peres J., Esperança P. (1998). Altered States of Consciousness: Psychoneuro physiology of Personalized Regressive and Experiential Imaginary Therapy. Aquém e Além do Cérebro.

[45] Hinterberger T., Schöner J., Halsband U. (2011). An Analysis of EEG State Transitions during Hypnosis Induction. Int. J. Clin. Exp. Hypn..

[46] Ryan R.M., Huta V. (2009). Wellness as healthy functioning or wellness as happiness: The importance of eudaimonic thinking (response to the Kashdan et al. and Waterman discussion). J. Posit. Psychol..

[47] Giuntoli L., Condini F., Ceccarini F., Huta V., Vidotto G. (2021). The different roles of hedonic and eudaimonic motives for activities in predicting functioning and well-being experiences. J. Happiness Stud..

[48] Pacheco A., Martins J.E., Simões M. (2019). Mental health promotion in the community: Conceptual analysis. Cad. Saúde.

[49] Hipólito I., Martins J. (2017). Mind-life continuity: A qualitative study of conscious experience. Prog. Biophys. Mol. Biol..

[50] Chiappelli F., Iribarren F.J., Prolo P. (2006). Salivary biomarkers in psychobiological medicine. Bioinformation.

[51] Jabeen R., Payne D., Wiktorowicz J., Mohammad A., Petersen J. (2006). Capillary electrophoresis and the clinical laboratory. Electrophor..

[52] Greenfield S.A., Collins T.F., Laureys S. (2005). A neuroscientific approach to consciousness. Progress in Brain Research.

[53] Mitra D., Chaudhary P., Verma D., Khoshru B., Senapati A., Mahakur B., Panneerselvame P., Das Mohapatraa P.K., Anđelković S. (2021). Bioinformatics’ Role in Studying Microbe-Mediated Biotic and Abiotic Stress Tolerance. Microbial Management of Plant Stresses.

[54] Lanktree M.B., Hassell R.G., Lahiry P., Hegele R.A. (2010). Phenomics: Expanding the role of clinical evaluation in genomic studies. J. Investig. Med..

[55] Martins J.E., Simões M., Ferreira H., Tavares V., Brito J., Carvalho L.X., Carvalho E.N., Castelo-Branco M. (2016). Self-reflexive consciousness: A model for the experimental use of neurofeedback in sensorial immersion in a center for consciousness knowledge. Exp. Pathol. Health Sci. Res. Clin. Teach. Soc..

[56] Wormwood K.L., Aslebagh R., Channaveerappa D., Dupree E.J., Borland M.M., Ryan J.P., Darie C.C., Woods A.G. (2015). Salivary proteomics and biomarkers in neurology and psychiatry. PROTEOMICS-Clin. Appl..

[57] Kendall P.C., Hollon S.D. (2013). Cognitive-Behavioral Interventions: Theory, Research, and Procedures.

[58] Norris L.A., Rabner J.C., Mennies R.J., Olino T.M., Kendall P.C. (2021). Increased self-reported reward responsiveness predicts better response to cognitive behavioral therapy for youth with anxiety. J. Anxiety Disord..

[59] Varela F.J. (1997). Sleeping, Dreaming, and Dying: An Exploration of Consciousness with the Dalai Lama. Mind and Life.

[60] Brockman J. (1996). Third Culture: Beyond the Scientific Revolution.

[61] Dictionary M.W. http://www.mw.com/home.htm.

[62] Thompson E. (2014). Waking, Dreaming, Being: Self and Consciousness in Neuroscience, Meditation, and Philosophy.

[63] Dunne J.D., Thompson E., Schooler J. (2019). Mindful meta-awareness: Sustained and non-propositional. Curr. Opin. Psychol..

[64] Thompson E., Stapleton M. (2009). Making Sense of Sense-Making: Reflections on enactive and extended mind theories. Topoi.

[65] Crane R. (2017). Mindfulness-Based Cognitive Therapy: Distinctive Features.

[66] Thompson E. (2010). Mind in Life: Biology, Phenomenology, and the Sciences of Mind.

[67] Best J.W., Kahn J.V. (2014). Research in Education.

[68] Biundo R., Weis L., Facchini S., Formento-Dojot P., Vallelunga A., Pilleri M., Antonini A. (2014). Cognitive profiling of Parkinson disease patients with mild cognitive impairment and dementia. Parkinsonism Relat. Disord..

[69] Britton W.B., Lindahl J.R., Cahn B.R., Davis J.H., Goldman R.E. (2014). Awakening is not a metaphor: The effects of Buddhist meditation practices on basic wakefulness. Ann. N. Y. Acad. Sci..

[70] Matthews P.M., Edison P., Geraghty O.C., Johnson M.R. (2014). The emerging agenda of stratified medicine in neurology. Nat. Rev. Neurol..

[71] Schumann G., Binder E.B., Holte A., de Kloet E.R., Oedegaard K.J., Robbins T.W., Walker-Tilley T.R., Bitter I., Browng V.J., Ciccocioppo R. (2014). Stratified medicine for mental disorders. Eur. Neuropsychopharmacol..

[72] Bearden C.E., Winkler A., Karlsgodt K.H., Bilder R. (2016). Cognitive Phenotypes and Endophenotypes: Concepts and Criteria. Neurophenotypes.

[73] Bearden C., Sun D., Lin A., Ching C., Jacquemont S., Moreau C., Villalon J. (2019). 250. Gene Dosage Effects on Neurobehavioral Phenotypes and Development: Relevance to Idiopathic Neuropsychiatric Disorders. Biol. Psychiatry.

[74] Britton W.B. (2019). Can mindfulness be too much of a good thing? The value of a middle way. Curr. Opin. Psychol..

[75] Martini A., Weis L., Schifano R., Pistonesi F., Fiorenzato E., Antonini A., Biundo R. (2020). Differences in cognitive profiles between Lewy body and Parkinson’s disease dementia. J. Neural Transm..

[76] Zhang Y., Hedo R., Rivera A., Rull R., Richardson S., Tu X.M. (2019). Post hoc power analysis: Is it an informative and meaningful analysis?. Gen. Psychiatry.

[77] Dahiru T., Aliyu A., Kene T.S. (2006). Statistics in medical research: Misuse of sampling and sample size determination. Ann. Afr. Med..

[78] Mohr P.J., Taylor B.N., Newell D.B. (2008). CODATA recommended values of the fundamental physical constants: 2006. J. Phys. Chem. Ref. Data.

[79] Sullivan G.M., Feinn R. (2012). Using effect size—Or why the P value is not enough. J. Grad. Med. Educ..

[80] Trzepacz P.T., Baker R.W. (1993). The Psychiatric Mental Status Examination.

[81] Taylor F.K. (1967). The role of phenomenology in psychiatry. Br. J. Psychiatry.

[82] Owen G., Harland R. (2006). Theme issue on phenomenology and psychiatry for the 21st century. Taking phenomenology seriously. Schizophr. Bull..

[83] Berrios G.E. (1989). What is phenomenology? A review. J. R. Soc. Med..

[84] Simões M., Polónio P., Von Arx S., Staub S., Dittrich A. (1986). Estudo Internacional sobre Estados de Consciência Alterados (ISASC): Resultados em Portugal. Psicologia.

[85] Pais-Ribeiro J.L. (2012). Validação transcultural da escala de felicidade subjectiva de Lyubomirsky e Lepper. Psicol. Saúde Doenças.

[86] Gregório S., Gouveia J.P. (2011). Facetas de mindfulness: Características psicométricas de um instrumento de avaliação. Psychologica.

[87] Neto F. (1986). Escala de consciência de si próprio: Adaptação portuguesa. Cadernos de Consulta Psicológica.

[88] Dittrich A. (1998). The standardized psychometric assessment of altered states of consciousness (ASCs) in humans. Pharmacopsychiatry.

[89] Sims A. (1988). Symptoms in the Mind: An Introduction to Descriptive Psychopathology.

[90] Fish F.J., Casey P.R., Kelly B. (2007). Fish’s Clinical Psychopathology: Signs and Symptoms in Psychiatry.

[91] Schacter D., Gilbert D., Wegner D., Hood B.M. (2011). Psychology: European Edition.

[92] Lyubomirsky S., Lepper H.S. (1999). A measure of subjective happiness: Preliminary reliability and construct validation. Soc. Indic. Res..

[93] Revord J., Sweeny K., Lyubomirsky S. (2021). Categorizing the function of positive emotions. Curr. Opin. Behav. Sci..

[94] Giannini A.J. (1986). The Biological Foundations of Clinical Psychiatry.

[95] Scheier M.F., Carver C.S. (1985). The Self-Consciousness Scale: A Revised Version for Use with General Populations1. J. Appl. Soc. Psychol..

[96] Erikson E.H. (1994). Insight and Responsibility.

[97] Martin W., Hickerson R. (2013). Mental capacity and the applied phenomenology of judgment. Phenomenol. Cogn. Sci..

[98] Quee P.J., van der Meer L., Bruggeman R., de Haan L., Krabbendam L., Cahn W., Mulder N.C.L., Wiersma D., Aleman A. (2011). Insight in psychosis: Relationship with neurocognition, social cognition and clinical symptoms depends on phase of illness. Schizophr. Bull..

[99] Studerus E., Gamma A., Vollenweider F.X. (2010). Psychometric evaluation of the altered states of consciousness rating scale (OAV). PLoS ONE.

[100] Dittrich A. (1996). Ätiologie-Unabhängige Strukturen Veränderter Wachbewußtseinszustände: Ergebnisse Empirischer Untersuchungen über Halluzinogene I. und II. Ordnung, Sensorische Deprivation, Hypnagoge Zustände, Hypnotische Verfahren Sowie Reizüberflutung; 119 Tabellen.

[101] Gouzoulis-Mayfrank E., Heekeren K., Thelen B., Lindenblatt H., Kovar K.A., Sass H., Geyer M.A. (1998). Effects of the hallucinogen psilocybin on habituation and prepulse inhibition of the startle reflex in humans. Behav. Pharmacol..

[102] Vollenweider F.X., Csomor P.A., Knappe B., Geyer M.A., Quednow B.B. (2007). The effects of the preferential 5-HT2A agonist psilocybin on prepulse inhibition of startle in healthy human volunteers depend on interstimulus interval. Neuropsychopharmacology.

[103] Vollenweider F.X., Geyer M.A. (2001). A systems model of altered consciousness: Integrating natural and drug-induced psychoses. Brain Res. Bull..

[104] Geyer M.A., Vollenweider F.X. (2008). Serotonin research: Contributions to understanding psychoses. Trends Pharmacol. Sci..

[105] Osman A., Lamis D.A., Bagge C.L., Freedenthal S., Barnes S.M. (2016). The mindful attention awareness scale: Further examination of dimensionality, reliability, and concurrent validity estimates. J. Personal. Assess..

[106] Hart W., Tortoriello G.K., Richardson K. (2019). Profiling public and private self-consciousness on self-presentation tactic use. Personal. Individ. Differ..

[107] Richardson K., Hart W., Tortoriello G.K., Breeden C.J. (2021). An interaction model for the role of self-evaluations and antagonistic pursuits in subjective well-being. Br. J. Psychol..

[108] Kavanagh J., Oliver S., Lorenc T., Caird J., Tucker H., Harden A., Greaves A., Thomas J., Oakley A. (2009). School-based cognitive-behavioural interventions: A systematic review of effects and inequalities. Health Sociol. Rev..

[109] Dunning D.L., Griffiths K., Kuyken W., Crane C., Foulkes L., Parker J., Dalgleish T. (2019). Research Review: The effects of mindfulness-based interventions on cognition and mental health in children and adolescents–a meta-analysis of randomized controlled trials. J. Child Psychol. Psychiatry.

[110] Culbert T. (2017). Perspectives on technology-assisted relaxation approaches to support mind-body skills practice in children and teens: Clinical experience and commentary. Children.

[111] Khema A., Brasington L., Heinegg P. (2001). Visible Here and Now: The Buddha’s Teachings on the Rewards of Spiritual Practice.

[112] Leung M.K., Lau W.K., Chan C.C., Wong S.S., Fung A.L., Lee T.M. (2018). Meditation-induced neuroplastic changes in amygdala activity during negative affective processing. Soc. Neurosci..

[113] Cotier F.A., Zhang R., Lee T.M. (2017). A longitudinal study of the effect of short-term meditation training on functional network organization of the aging brain. Sci. Rep..

[114] Xu M., Purdon C., Seli P., Smilek D. (2017). Mindfulness and mind wandering: The protective effects of brief meditation in anxious individuals. Conscious. Cogn..

[115] Hoge E.A., Guidos B.M., Mete M., Bui E., Pollack M.H., Simon N.M., Dutton M.A. (2017). Effects of mindfulness meditation on occupational functioning and health care utilization in individuals with anxiety. J. Psychosom. Res..

[116] Simões M., Oliveira M., Marujo H.A., Neto L.M., Ribeiro J.A., Marto J., Simões M. (2013). Psicoterapia Breve Trajetória de Vida—A Hipnose Clínica do Individual ao Grupal na Comunidade Positiva. Hipnose Clínica.

[117] Damasio A., Meyer K. (2009). Consciousness: An overview of the phenomenon and of its possible neural basis. The Neurology of Consciousness: Cognitive Neuroscience and Neuropathology.

[118] Gallagher S. (2005). How the Body Shapes the Mind.

[119] Wager T.D., Atlas L.Y. (2015). The neuroscience of placebo effects: Connecting context, learning and health. Nat. Rev. Neurosci..

[120] Carvalho G.B., Damasio A. (2021). Interoception and the origin of feelings: A new synthesis. BioEssays.

[121] Laureys S., Gosseries O., Tononi G. (2015). The Neurology of Consciousness: Cognitive Neuroscience and Neuropathology.

[122] Demertzi A., Tagliazucchi E., Dehaene S., Deco G., Barttfeld P., Raimondo F., Martial C., Fernández-Espejo D., Rohaut B., Voss H.U. (2019). Human consciousness is supported by dynamic complex patterns of brain signal coordination. Sci. Adv..

[123] Kirsch I., Kong J., Sadler P., Spaeth R., Cook A., Kaptchuk T.J., Gollub R. (2014). Expectancy and conditioning in placebo analgesia: Separate or connected processes?. Psychol. Conscious. Theory Res. Pract..

[124] Kube T., Kirsch I., Glombiewski J.A., Herzog P. (2022). Can placebos reduce intrusive memories?. Behav. Res. Ther..

[125] Veenhoven R., Dutt A.K., Radcliff B. (2009). Chapter 3: How Do We Assess How Happy We Are? Tenets, Implications and Tenability of Three Theories. Happiness, Economics and Politics.

[126] Jack A.I. (2003). Trusting the Subject? The Use of Introspective Evidence in Cognitive Science Volume.

[127] Bossuyt X. (2006). Advances in serum protein electrophoresis. Adv. Clin. Chem..

[128] Kimman J., Bossuyt X., Blockmans D. (2018). Prognostic value of cryoglobulins, protein electrophoresis, and serum immunoglobulins for lymphoma development in patients with Sjögren’s syndrome. A retrospective cohort study. Acta Clin. Belg..

[129] Woods B., Aguirre E., Spector A.E., Orrell M. (2012). Cognitive stimulation to improve cognitive functioning in people with dementia. Cochrane Database Syst. Rev..

[130] Whitfield T., Barnhofer T., Acabchuk R., Cohen A., Lee M., Schlosser M., Arenaza-Urquijo E.M., Böttcher A., Britton W., Coll-Padros N. (2022). The Effect of Mindfulness-based Programs on Cognitive Function in Adults: A Systematic Review and Meta-analysis. Neuropsychol Rev..

[131] Bayne T., Cleeremans A., Wilken P. (2014). The Oxford Companion to Consciousness.

[132] Cleeremans A., Tallon-Baudry C. (2022). Consciousness matters: Phenomenal experience has functional value. Neurosci. Conscious..

[133] Stuyck H., Cleeremans A., Van den Bussche E. (2022). Aha! under pressure: The Aha! experience is not constrained by cognitive load. Cognition.

[134] Seth A.K., Bayne T. (2022). Theories of consciousness. Nat. Rev. Neurosci..

[135] VandenBos G.R. (2013). APA Dictionary of Clinical Psychology.

[136] De Jaegher H., Pieper B., Clénin D., Fuchs T. (2017). Grasping intersubjectivity: An invitation to embody social interaction research. Phenomenol. Cogn. Sci..

[137] Di Paolo E.A., De Jaegher H. (2022). Enactive ethics: Difference becoming participation. Topoi.

[138] Klein S.B., Loftus J., Srull T.K., Wyer R.S. (2015). The mental representation of trait and autobiographical knowledge about the self. The Mental Representation of Trait and Autobiographical Knowledge about the Self: Advances in Social Cognition.

[139] Husserl E., Biemel M. (1952). Ideen zu Einer Reinen Phänomenologie und Phänomenologischen Philosophie. Zweites Buch: Phänomenologische Untersuchungen zur Konstitution.

[140] Merleau-Ponty M. (1976). Phénoménologie de la Perception (1945).

[141] Gallagher S., Zahavi D. (2020). The Phenomenological Mind.

[142] Osborn M., Smith J.A. (2006). Living with a body separate from the self. The experience of the body in chronic benign low back pain: An interpretative phenomenological analysis. Scand. J. Caring Sci..

[143] Mozo-Dutton L., Simpson J., Boot J. (2012). MS and me: Exploring the impact of multiple sclerosis on perceptions of self. Disabil. Rehabil..

[144] Smith J.A., Nizza I.E. (2022). Essentials of Interpretative Phenomenological Analysis.

[145] Hipólito I., Martins J.E., Hipólito I., Gonçalves J., Pereira J. (2018). A “Second-Person” Model to Anomalous Social Cognition. Schizophrenia and Common Sense. Studies in Brain and Mind.

[146] Buckner R.L., Snyder A.Z., Shannon B.J., LaRossa G., Sachs R., Fotenos A.F., Sheline Y.I., Klunk W.E., Mathis C.A., Morris J.C. (2005). Molecular, structural, and functional characterization of Alzheimer’s disease: Evidence for a relationship between default activity, amyloid, and memory. J. Neurosci..

[147] Fredrickson B.L., Grewen K.M., Coffey K.A., Algoe S.B., Firestine A.M., Arevalo J.M., Ma J., Cole S.W. (2013). A functional genomic perspective on human well-being. Proc. Natl. Acad. Sci. USA.

[148] Van Cappellen P., Catalino L.I., Fredrickson B.L. (2020). A new micro-intervention to increase the enjoyment and continued practice of meditation. Emotion.

[149] Correia M.J., Martins J.E., Esteves E., Fernandes M., Cruz I., Rosa N., Barros M. Bringing Saliva into Research—Molecular Typing of Individuals. Proceedings of the Science 2017: Science and Technology Foundation.

[150] Martins J.E., Simões J., Simões M. (2022). Case Report Form (CRF) applied after a Cognitive Behavioral Intervention and Mental Status Examination Of Neurotypical Young Adults.

[151] Martins J.E., Simões J., Simões M. (2022). Verbal Protocol of the Experimental Procedure Used in "Pre-Molecular Assessment of Self-Processes in Neurotypical Subjects Using a Single Cognitive Behavioral Intervention Evoking Autobiographical Memory".

